# Federated learning inspired Antlion based orchestration for Edge computing environment

**DOI:** 10.1371/journal.pone.0304067

**Published:** 2024-06-04

**Authors:** Madhusudhan H. S., Punit Gupta

**Affiliations:** 1 Department of Computer Science and Engineering, Vidyavardhaka College of Engineering, Mysuru, Karnataka, India; 2 University College Dublin, Dublin, Ireland; 3 Manipal University Jaipur, Jaipur, Rajasthan, India; Chitkara University, INDIA

## Abstract

Edge computing is a scalable, modern, and distributed computing architecture that brings computational workloads closer to smart gateways or Edge devices. This computing model delivers IoT (Internet of Things) computations and processes the IoT requests from the Edge of the network. In a diverse and independent environment like Fog-Edge, resource management is a critical issue. Hence, scheduling is a vital process to enhance efficiency and allocation of resources properly to the tasks. The manuscript proposes an Artificial Neural Network (ANN) inspired Antlion algorithm for task orchestration Edge environments. Its aim is to enhance resource utilization and reduce energy consumption. Comparative analysis with different algorithms shows that the proposed algorithm balances the load on the Edge layer, which results in lower load on the cloud, improves power consumption, CPU utilization, network utilization, and reduces average waiting time for requests. The proposed model is tested for healthcare application in Edge computing environment. The evaluation shows that the proposed algorithm outperforms existing fuzzy logic algorithms. The performance of the ANN inspired Antlion based orchestration approach is evaluated using performance metrics, power consumption, CPU utilization, network utilization, and average waiting time for requests respectively. It outperforms the existing fuzzy logic, round robin algorithm. The proposed technique achieves an average cloud energy consumption improvement of 95.94%, and average Edge energy consumption improvement of 16.79%, 19.85% in average CPU utilization in Edge computing environment, 10.64% in average CPU utilization in cloud environment, and 23.33% in average network utilization, and the average waiting time decreases by 96% compared to fuzzy logic and 1.4% compared to round-robin respectively.

## 1. Introduction

Cloud computing has emerged as a significant trend for storing information, transferring, and controlling computing resources in geographically distributed data centers over the last few years. Cloud computing, on the other hand, confronts rising challenges like high uncertainty in communication latency, end devices network load, privacy gaps and costs associated with head-on connections [1, 2]. Furthermost, the issues are because of the substantial physical space between the data centers of cloud service providers and end users. This geographical distance incurs extreme latency and poor service quality (QoS). These challenges act as obstacles for time-sensitive application queries, making real-time processing and delivering rapid response to end users difficult [1].

In 2014, Cisco introduced Fog computing as an extension of the cloud computing paradigm to the network Edge. Fog-Edge is a virtual platform that connects end-users of IoT devices, providing computing, storage, and network services [3]. The primary goal of Fog computing is to achieve ubiquitous distributed computing by decentralizing computing and integrating cloud data centers with heterogeneous Edge devices [4, 5]. Moreover, Fog base stations can handle lightweight or time-sensitive tasks in IoT environments.

Even though Fog computing is an efficient technique for handling cloud computing issues, there are still some issues that need to be addressed. Most importantly, the Fog layer’s network, storage resources and computational must be managed by a smart distributed system at the Edge. Anomalies induced by the relationship among forthcoming requests & confined Fog node resources currently produce many barriers to appropriate resource allocation decision-making.

Likewise, if each query is dispatched from the IoT layer to cloud, potential to respond to low latency requests is jeopardized. While reacting to all queries with minimal latency, Fog computing faces resource scarcity issues. As a result, smart management of resources at the Fog layer could be a viable result to this hitch.

**Fig 1:**
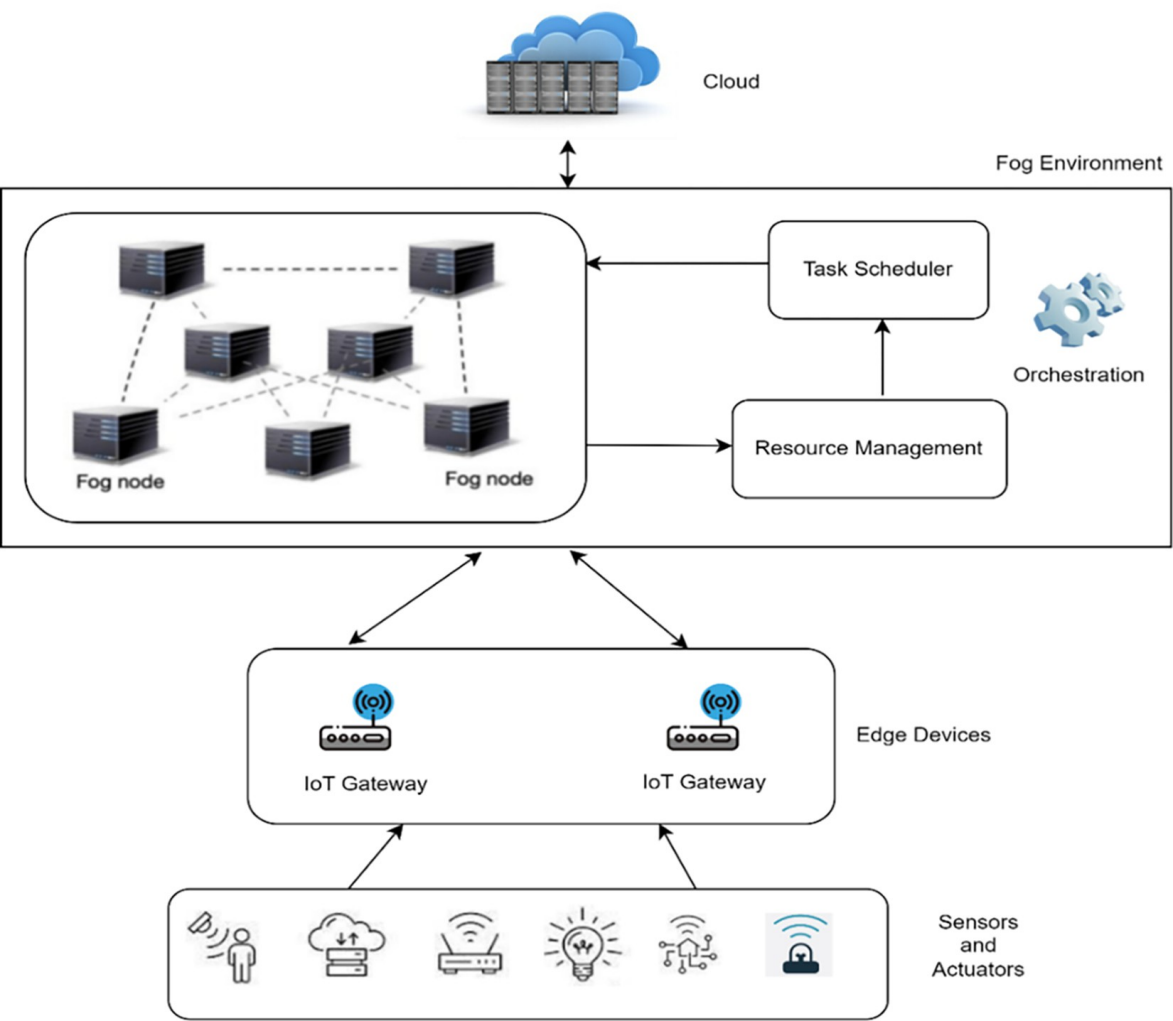
System architecture.

Fig 1 depicts the architecture of Fog-Edge computing. It consists of several layers like cloud, Fog environment, Edge devices, sensors, and actuators. Edge devices send the requests that need to be processed in the Fog environment. The task orchestration comprises resource management and task scheduler. Here, resource management is responsible for monitoring the status of all the available resources in a Fog environment. The task scheduler is developed using the Antlion algorithm.

Main contributions of the proposed work are as follows:

1. Design of Machine learning inspired Antlion algorithm for solving task scheduling problems in the Fog-Edge environment
2. To minimize energy consumption, makespan and maximize resource utilization.
3. Machine learning-inspired decentralized orchestration algorithm
4. To monitor resources available in the Edge environment.

The manuscript is divided into six sections. Section 2 showcases the existing works, section 3 covers the proposed model, and Section 4 shows the proposed model for task scheduling. Section 5 covers the experimental results of the cloud and Edge scenarios using performance metrics energy, time, and resource utilization (CPU utilization). A detailed description of experimental results is included in the sub-section 5.1 to sub section 5.5.

## 2. Related work

This section presents some of the prominent meta-heuristic approaches used in Fog environments. Table 1 depicts the studies and their parameters. Also, techniques are categorized into Nature/Bio Inspired methods & Metaheuristic approaches and Machine Learning/Deep Learning techniques.

**Table 1:** Classification of different approaches in existing literature.

Reference	Algorithm Used	Parameters					Missing Parameters
		Time	Resource Utilization	Energy Consumption	Number of tasks completed	Operating Cost/Load Balancing	
6	PSO	✓				✓	Resource Utilization, energy consumption, tasks completed
7	GA-PSO	✓		✓			Resource Utilization, tasks completed
8	Firework algorithm	✓				✓	Resource Utilization, energy consumption, tasks completed
9	GA	✓					Resource Utilization, energy consumption, tasks completed
10	TCaS	✓				✓	Resource Utilization, energy consumption, tasks completed
11	Harris Hawk Optimization	✓					Resource Utilization, energy consumption
12	Fireworks algorithm and Earliest Finish Time	✓			✓	✓	Resource Utilization, energy consumption
13	Cuckoo Search	✓	✓	✓	✓		
14	Grey Wolf Optimization	✓		✓			Number of tasks completed
17	Reinforcement Learning	✓				✓	Resource Utilization, energy consumption
21	Collaborative Machine Learning	✓	✓	✓			

### 2.1. Nature/bio inspired methods & metaheuristic approaches

EPSO, a resource scheduling technique proposed by Narayana et al. [6], was implemented in a Fog environment. Authors used the PSO method in conjunction with the proximal gradient method to ease the convex problems. To converge the local and global solutions, the developed method employs additional gradient parameters. To demonstrate the effectiveness of the developed method, total cost and makespan metrics were compared. The developed method shortens the execution time of many tasks at the expense of increased energy consumption.

Yadav et al. [7] proposed a service distribution method in Fog environment that is a blend of PSO and GA methods to achieve ideal service distribution. The proposed technique lays services on Fog nodes to attain the appropriate feasible execution while minimizing the time and energy consumption for applications of IoT.

The fireworks algorithm task scheduling technique was introduced in [8]. Authors demonstrated a mechanism for detecting fireworks explosions. This paper also used the task clustering method. Memory and CPU utilization metrics were used in their load utilization model without regard for cost. Aburukba et al. [9] presented a model scheduling application in IoT via integer programming, with the goal of minimizing service request latency. Authors made use of the genetic algorithm to schedule IoT applications and compared the method to priority-strict queuing, round-robin, and waited-fair queuing in terms of latency parameter. It is observed that the proposed method also demonstrated substantial progress in achieving query deadlines by up to 31%.

In their work [10], Yang et al. proposed Fog simulator and a task scheduling method that considers execution time and resource costs. The work proposes a multi-objective evolutionary method that changes the neighborhood based on the present Fog computing scenario of the task scheduling group to distribute the current job scheduling group more efficiently. The authors demonstrated how their approach reduces load, job execution time, and resource cost. However, unlike other analyzed scheduling systems, the availability of Fog suppliers was not considered in this work.

Authors in [11] presented Hidden Morkov Model and Harris Hawk Optimization algorithms for predicting each Fog provider’s availability and scheduling of tasks respectively. Proposed work reduces offloaded tasks, SLA violation and deadline-missed workflows.

Yadav et al. [12] proposed fireworks algorithm to design scheduling algorithm in Fog environment. Authors have also considered immediate finish time, which is a heuristic approach. The hybrid method intended to speed up the search by utilizing both solution space exploitation and exploration. The aim of this work is to reduce cost and makespan and increase throughput. Cuckoo search was proposed [13] to address placement in IoT service. This multi objective work focuses on various metrics like energy consumption, response time, throughput, and delay. Both Quality of Service and optimization resource utilization in Fog environment is addressed in this work.

A multi objective grey wolf optimization algorithm is presented by Faten A. Saif et al. [14]. The proposed work plays a vital role in distributing tasks in Fog-cloud system. Evaluation result shows the effectiveness of grey wolf algorithm in reducing energy consumption and delay. In [15], authors propose an autonomic IoT service placement technique based on the grey wolf optimisation scheme to deploy IoT applications on Fog nodes, improving system performance while taking execution costs into account. Furthermore, the autonomic ideas aid in the development of an auto management system that better matches the dynamic behaviour of the Fog environment.

### 2.2. Machine learning/deep learning techniques

The learning automata approach is proposed by the authors in [16] as an effective dynamic service provisioning mechanism to decide whether to release or deploy IoT applications across the heterogeneous and dynamic Fog infrastructure. Additionally, to install the IoT applications on the Fog infrastructure, the authors created an autonomous dynamic service provisioning manager (DSPM) that adheres to a self-management control loop.

In [17], a Deep Reinforcement Learning-based IoT application scheduling method named DRLIS is presented to balance the load on the Edge/Fog servers and adaptively and efficiently optimize the response time of heterogeneous IoT applications. The authors developed an Edge-Fog-cloud integrated serverless computing system using the FogBus2 function-as-a-service architecture and DRLIS as a workable scheduler. Comprehensive experiment results demonstrate that DRLIS dramatically lowers the execution cost of Internet of Things applications. Load balancing, energy conservation, and scheduling demands have an impact on the Edge-Fog-cloud computing architecture’s performance and dependability. To overcome these difficulties, a reinforcement learning Fog scheduling technique is put out in [18]. Comparing the suggested approach to the current scheduling techniques, the testing findings show that it improves load balancing and reduces response time.

Zhang et al., [19] proposed a method for MTCN’s multi-target-aware dynamic resource scheduling that increases resource flexibility. The authors established multi-target-aware embedded limitations and considered differentiable QoS needs such as computing, storage, bandwidth, latency, and so forth. The authors also describe a scheduling network based on Deep Reinforcement Learning (DRL) that can effectively and scientifically interact with the MTCN environment.

Authors in [20] investigated resource auto-scaling in the Fog environment to prevent issues with over- and under-provisioning of Fog resources for time-varying demands from IoT devices. Deep learning was used as a solution to this issue. Additionally, to explain the interactions between the intended modules, IoT, Fog, and cloud devices for automatic resource scaling, a framework for resource auto-scaling based on the three-tier Fog architecture was suggested. In [21], authors suggest a resource allocation strategy using collaborative machine learning (CML) for SDN-enabled Fog computing. The resource allocation strategy for the SDN-enabled Fog computing environment is linked with the suggested CML model. The outcomes of the suggested approach are tested using FogBus and iFogSim, utilizing a variety of performance assessment measures such execution time, power consumption, latency, and bandwidth utilization.

Fuzzy logic with the K-Means clustering algorithm to efficiently arrange Fog nodes according to their workload patterns and resource attributes is presented in [22]. The suggested technique assigns jobs to Fog nodes dynamically by fusing fuzzy logic’s flexibility with Keans’ clustering powers. It describes how jobs may be intelligently assigned to Fog nodes by utilizing machine learning techniques, which will reduce execution time, response time, and network utilization.

To summarize, the survey reported in this section aims at scheduling/load balancing in Fog and Edge computing environments. However, most of them focus on optimization without considering resource utilization, energy and the number of tasks completed. The proposed work in this article focuses on multi-objective task scheduling algorithm using hybrid approach to improve overall performance in Fog-Edge computing.

## 3. Proposed model

**Fig 2:**
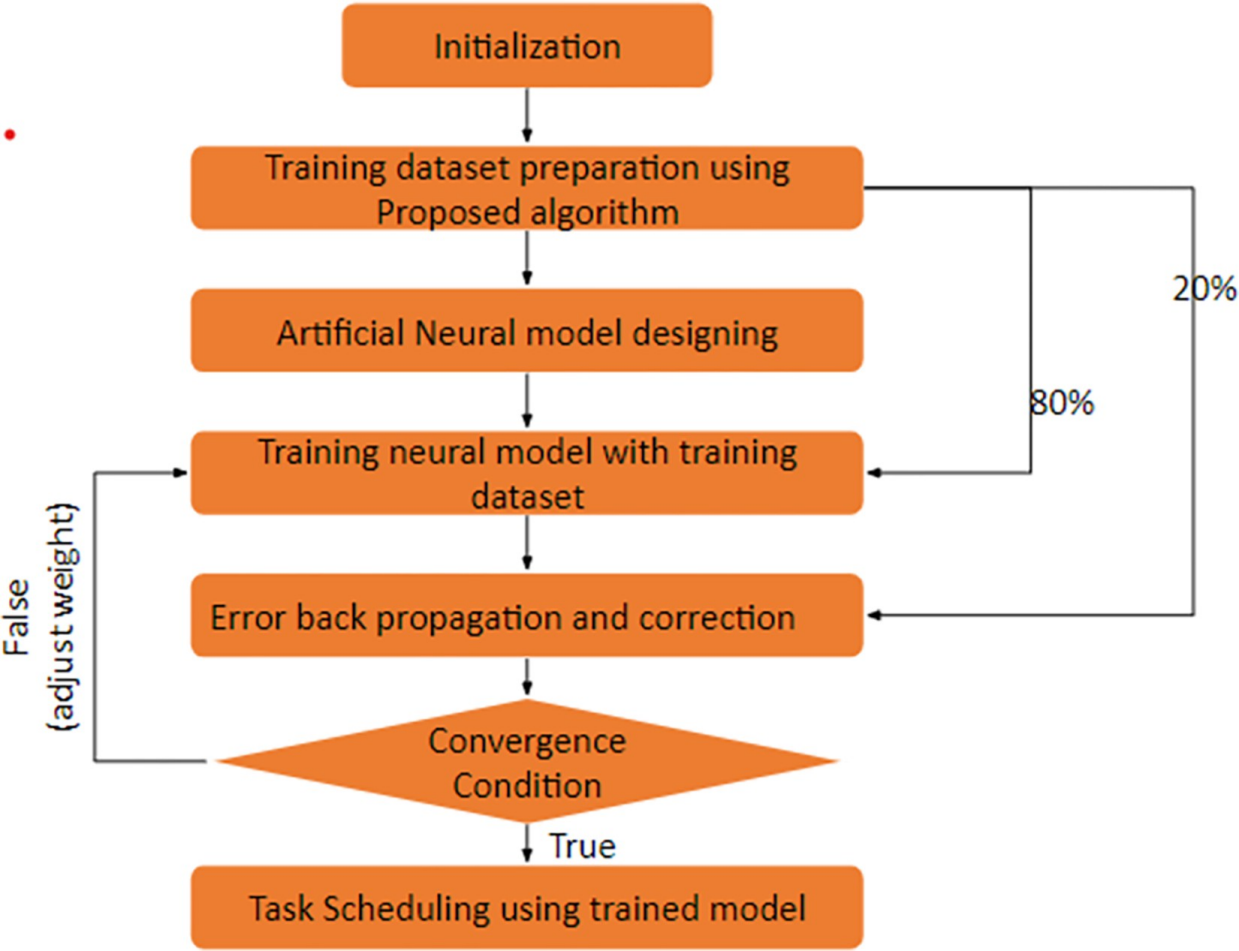
Proposed machine learning model.

This work presents an orchestration algorithm for Fog environment to optimize the resource allocation and minimize makespan in Fog environment. The aim of this work is to complete the tasks at Fog layer reducing load over the cloud. The ANN inspired Antlion algorithm is proposed to complete majority of tasks near to the user/client using intelligent orchestration algorithm. The Work aims to improve the total execution time and minimize load over the cloud server. The proposed model is aimed to design a trained machine learning model using artificial neural network which trained with Antlion optimizer for best optimization as shown in Fig 2. The trained model will act as decentralized unit which can be deployed in a distributed environment. The work is aimed to first generate an optimal schedule using an Antlion optimizer and further training it to ANN.

The proposed model is divided into various phases given below:

1. Initialization of ANN for Resource Allocation
2. Training dataset preparation using Antlion Intelligent Orchestration Technique
3. Machine learning model designing
4. Model training
5. Model correction
6. Resource Allocation in Fog Environment

### 3.1. Initialization of ANN for resource allocation

To initialize the artificial neural network for resource allocation in a Fog-Edge environment first, the problem needs to be solved using the ANN should be clearly defined. In this case, the problem is resource allocation in a Fog-Edge environment. This involves deciding which resources (e.g., CPU, memory, network bandwidth) should be allocated to which tasks or applications running on the Fog or Edge nodes. Next, the input and output variables of the ANN should be determined. The selection of input variables for resource allocation in a Fog-Edge environment may include workload on each Fog or Edge node, available resources on each node, and QoS requirements of each task or application. The output variable is the allocation of resources to each task or application.

### 3.2. Training dataset preparation using Antlion intelligent orchestration technique

The Antlion Intelligent Orchestration Technique (AIOT) is a swarm intelligence-based optimization algorithm that is suitable for resource allocation in a Fog-Edge environment. In this phase, a dataset is generated using Antlion optimization for task scheduling. Here the process has resources and a list of tasks which are fed to the Antlion optimizer to generate an optimal schedule with the least power and makespan. This outcome consists of a list of tasks and the resources allocated to them which can be Fog, Edge, or cloud resource. Each run generates an output representing the optimal allocation of resources for each task or application. The resulting input-output pairs can be saved as data points in the training dataset.

### 3.3. Antlion optimization algorithm

The proposed optimization model is a nature-inspired model. This model is chosen for optimization because this model is better optimized than other swarm-based models like ACO, PSO, Butterfly and many more in terms of optimization time and finding the global best solution. This model allows us to find the best optimal solution without sticking in local optimal solution which has least probability.

The mathematical model corresponding to each step is described in the paragraph that follows.

1. Position of each ants and Antlions in *A*_*ant*_ and *A*_*Antlion*_ matrices respectively. For modeling ant’s movement, Eq (1) is used:
$$M(s)=0,c\_sum(2rn(s_{1})-1),c\_sum(2rn(s_{1})-1),\ldots ,c\_sum(2rn(s_{max})-1)$$ (1)
  Where *c_sum* denotes the cumulative sum, *max* and *s* represent maximum number of iterations and steps of random walk respectively.
  *c_sum* and stochastic function *rn*(s) are expressed as:
$$c\_sum(2rn(s_{max})-1)=\sum_{i=1}^{n-1}2rn(s_{i})-1$$ (2)
$$rn(s)=\left\{ \begin{array}{c} 1\;if\;r>0.5\\ 0\;if\;r\leq 0.5 \end{array}\right.$$ (3)
  To keep ants’ movement within the search space, normalization of ant’s random walk is performed as:
$$n_{j}^{t}=\frac{\left(n_{j}^{t}-l_{j})\times (\alpha_{j}^{t}-\delta_{j}^{t}\right)}{\left(h_{j}-l_{j}\right)}+\delta_{j}^{t}$$ (4)
  where $n_{j}^{t}$ represents the j^th^ ant position at iteration *t*, *l*_*j*_ and *h*_*j*_ denotes smallest and largest random walk of j^th^ variable, $\delta_{j}^{t}$ and $\alpha_{j}^{t}$ are the lowest and highest of j^th^ variable in iteration t.
2. Ant entrapment:
  Ant entrapment in the Antlion pit is achieved by changing the random walks of ants.
$$\delta_{j}^{t}={Antlion}_{j}^{t}+\delta^{t}$$ (5)
$$\alpha_{j}^{t}={Antlion}_{j}^{t}+\alpha^{t}$$ (6)
  where *δ*^*t*^ and *α*^*t*^ denotes the lowest and highest values of all variables in iteration t, $\delta_{j}^{t}$ and $\alpha_{j}^{t}$ represents the lowest and highest of j^th^ ant, ${Antlion}_{j}^{t}$ denotes position of j^th^ Antlion selected at t^th^ iteration.
3. Trap construction:
  ALO employs the Roulette Wheel selection method to find better Antlions with higher fitness values. These fittest Antlions attract more ants. To simulate ant sliding the random walk of ants should be reduced as
$$\delta^{t}=\frac{\delta^{t}}{I},\;\alpha_{j}^{t}=\frac{\alpha_{j}^{t}}{I}$$ (7)
  where I is the ratio mentioned in [23].
4. Catching prey and rebuilding the trap:
  The fitness function is calculated. Eq (8) is adopted for this process:
$${Antlion}_{j}^{t}={Ant}_{i}^{t}\;if\;fit({Ant}_{i}^{t})>fit({Antlion}_{j}^{t})$$ (8)
  When the ants leave the search space, the boundary checking mechanism is applied. The ant must return to the search domain after leaving it.
5. Elitism:
  Elitism is performed to store or retain the fittest Antlion for the subsequent generations. The retained Antlions are considered elite. Elite affects the manoeuvre of all the ants through iterations along with the Antlion chosen by roulette wheel selection.
$${Ant}_{i}^{t}=\frac{R_{Ant}^{t}+R_{E}^{t}}{2}$$ (9)
  where $R_{Ant}^{t}$ represents the random number to represent ant path, $R_{E}^{t}$ denotes random path the elite at *t*^*th*^ iteration, and ${Ant}_{i}^{t}$ is the ant lion position.

#### 3.3.1 Pseudo code: Ant lion algorithm

1. **Initialization**: Population of ants = P, Number of Antlions = N, maximum epoch, or iteration = *max*, current iteration = *t*, Random walk = *R*. Initialize the solutions randomly (of both Antlions and ants)

2. Compute the fitness function of Antlions and ants.

3. Identify the best Antlion and consider it to be optimal or the *Elite*

4. **While *t<max* do**

5.  **for** each Antlion solution

6.   use Roulette Wheel to select an Antlion

7.   Slide ant towards selected Antlion using Eq (7)

8.   The position of the ant is updated using Eq (9)

9.  end **for**

10.  Compute the fitness of all ants

11.  Use Eq 8 to substitute Antlion

12.  If an Antlion fitness> elite, then update elite

13. End **While**

**Return** *Elite*

**Fig 3:**
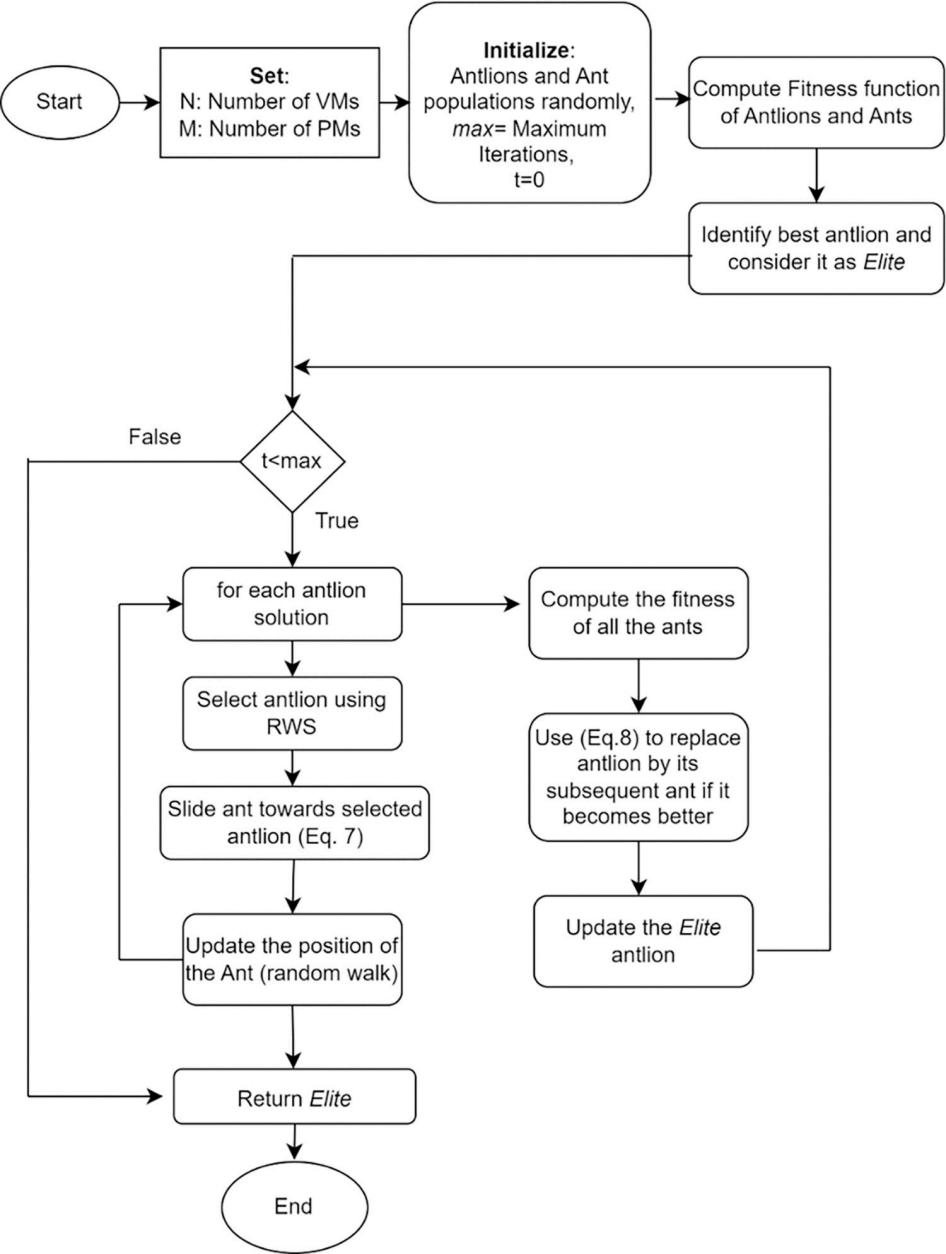
Antlion algorithm workflow.

The proposed Antlion algorithm process is described in Pseudo code and the workflow is described in Fig 3. Here, the population of solutions is initially seeded as ants on a search space. The position of ants and Antlions are stored as matrices. Fitness of Antlions, ants are computed, and the best Antlion is identified as Elite. Next, till the termination condition is met, for each Antlion solution, an Antlion is selected using roulette wheel selection. Subsequently, ants are made slide towards Antlion using the trap constructed and then position of the ant is updated. Fitness of all the ants is computed the Antlion will be replaced by ants if its fitness is higher and update the elite. Finally, once the termination condition is met, elite is returned as fittest Antlion.

Once the data has been generated, it should be preprocessed to ensure that it is in a suitable format for training the ANN. This could involve scaling the input variables to a common range, one-hot encoding categorical variables, and normalizing the output variables. The training dataset should be split into a training set and a validation set. To train the ANN, the training set is used to minimize the error between the actual and desired outputs, while the validation set is used to evaluate its performance. A suitable algorithm, such as backpropagation, can be used for this purpose. After training the ANN using the training dataset, it can then be used to allocate resources in a Fog-Edge environment based on the input variables. The output of the ANN represents the optimal allocation of resources for each task or application.

**Fig 4:**
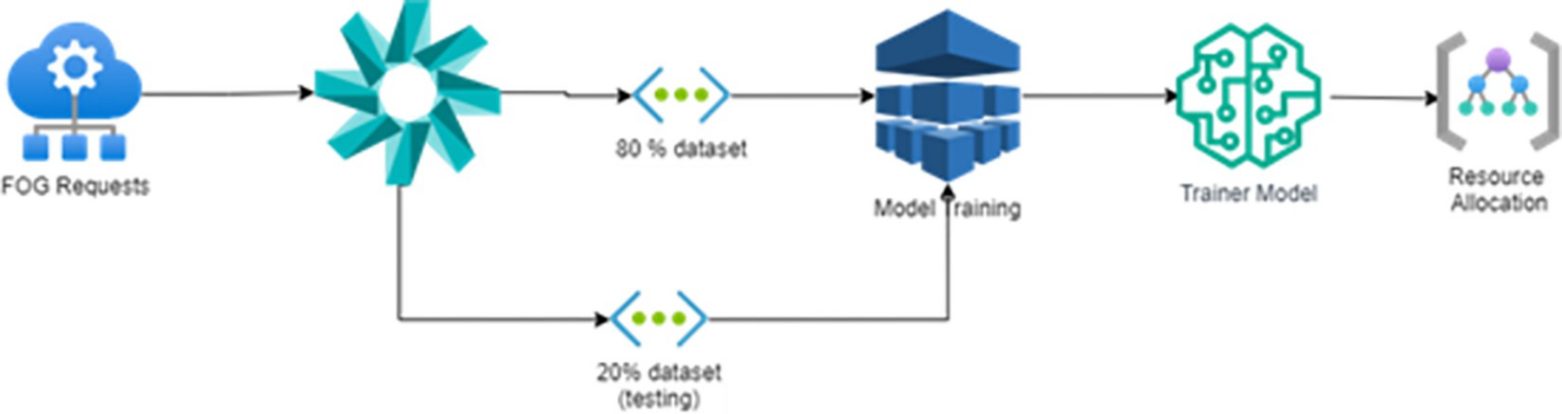
Flow of orchestration model.

Fig 4 shows the flow diagram of the proposed orchestration model for Fog-Edge computing environment. The key steps are defined properly using Input sequence of the Fog nodes and Edge nodes with optimal fitness function using Antlion technique. The offspring is used to train the neural network which provides the better results of the orchestration in a cloud and Edge computing environment.

### 3.4 Neural model designing

**Fig 5:**
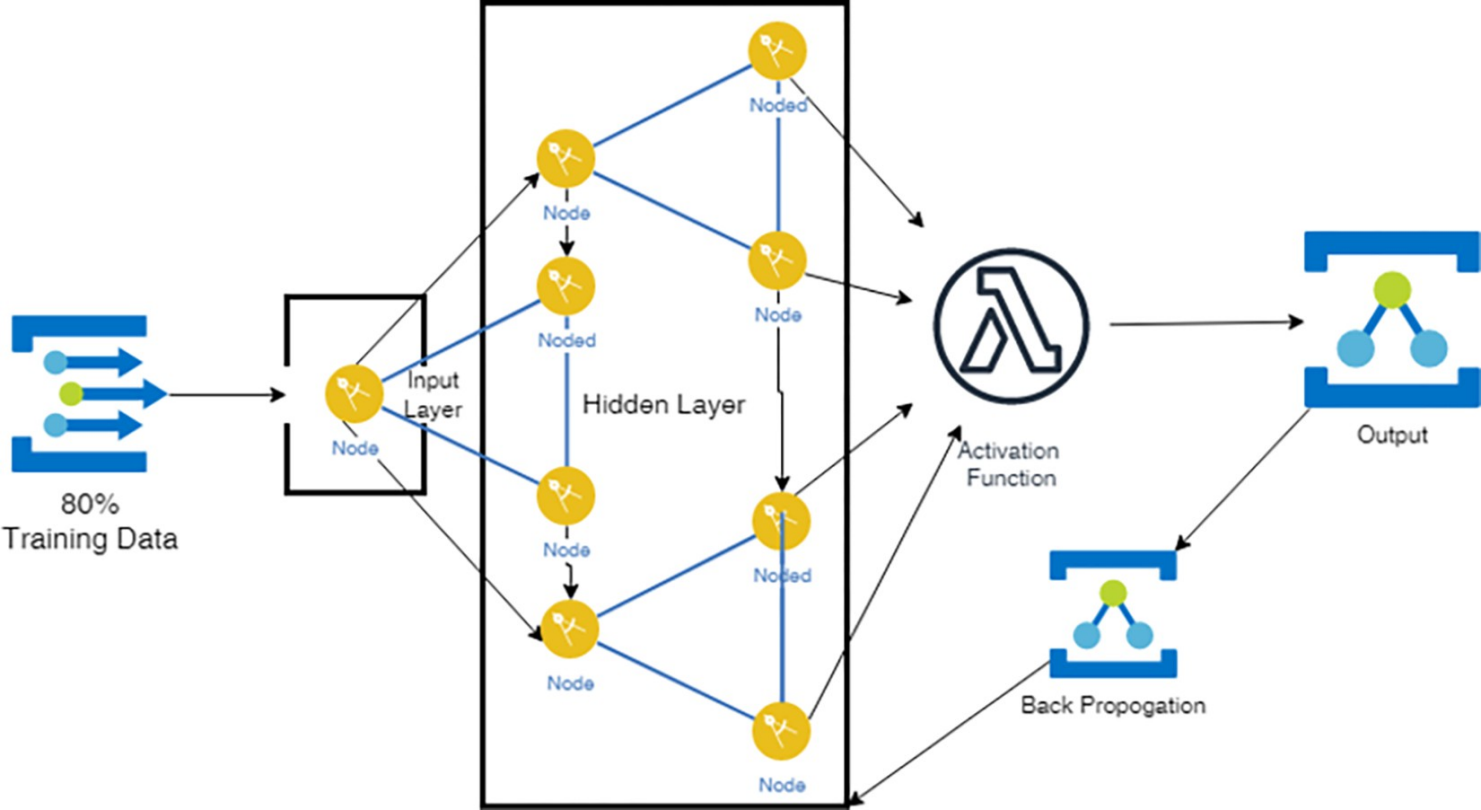
System architecture of feedforward neural network.

Fig 5 shows the architecture of the proposed ANN design, which consists of two input variables fed into the first hidden layer, comprised of four neurons. The outputs of the first hidden layer are then forwarded to the second hidden layer, which also consists of four neurons. Next, the outputs of the second hidden layer are passed to the third hidden layer, consisting of four neurons as well. Finally, the output of the third hidden layer is fed into the output layer, containing a single neuron. Each neuron in the hidden and output layers is associated with an activation function that transforms the sum of its weighted inputs into an output. The proposed ANN design includes several activation functions, such as the sigmoid, ReLU, and Leaky ReLU functions. The ANN model consists of an Input layer, 3 dense layers with activation function ReLu, Leaky ReLU and Softmax and the last layer being the output layer as shown in Table 2. Where ADAM is used as an optimizer to optimize the ANN model.

**Table 2:** Configuration of proposed neural network.

Number of layers	5
Number of epochs	150
Batch Size	10
Number of Neurons in dense layer initially	Equal to number of VM’s
Optimizer	adam
Loss Function	Categorical_Crossentropy

### 3.5. Neural model training

During training, the neural model iteratively adjusts the weights of the connections between neurons to improve its ability to predict the output given the input. This phase adjusts the weights of interconnected neurons in the network to predict the expected output using activation function. Once the model is trained, it can be used to make predictions on new input data that it has not seen before.

### 3.6. Error back propagation and correction

The process involves propagating the error backwards through the network from the output layer to the input layer. The correction of the weights during the backpropagation process is done to minimize the error. The optimization algorithm used during training ensures that the weights are updated in a way that is efficient and effective at minimizing the loss function.

The basic equation for the error backpropagation algorithm is:

δ_j=f'(z_j)*∑_kw_jkδ_k (10)

where δ_j is the error term for neuron j in the network shown in Fig 5, f’(z_j) is the derivative of the activation function of neuron j with respect to its input, w_jk is the weight of the connection between neuron j and neuron k in the network, δ_k is the error term for neuron k in the network, ∑_k w_jk δ_k is the sum of the weighted error terms for all neurons k that are connected to neuron j.

Once the error terms have been calculated for each neuron in the network, the weights are adjusted using the following equation:

Δw_jk=α*δ_k*a_j (11)

Where, Δw_jk is the change in weight of the connection between neuron j and neuron k, α is the learning rate which determines the step size of the weight update, δ_k is the error term for neuron k, a_j is the activation of neuron j which is the output of neuron j after applying its activation function to its input. The weights are updated iteratively using this equation until the error of the network is minimized.

## 4. Resource allocation on Fog-Edge environment

In a Fog environment, resource allocation entails distributing processing and storage capacity across several apps operating on Fog nodes. Because of how dynamic the Fog environment is and how quickly resources might vary in capacity and availability, this is a difficult problem.

One approach to resource allocation in a Fog environment is to use an artificial neural network (ANN) with Antlion intelligent orchestration technique. The ANN has been trained to manage resources using the Antlion intelligent orchestration technique, an optimization algorithm inspired by nature. Multiple input nodes, each representing a distinct characteristic like CPU, memory, and network bandwidth utilization, can be used while designing the artificial neural network. The allocation decision, such as which Fog node to assign the application to and how much resource to allocate to it, can be represented by the output node of the artificial neural network. The ANN gains knowlEdge through training by analyzing past data on resource allocation and use choices. The weights and biases of the ANN are optimized using the Antlion intelligent orchestration technique to reduce the error between the predicted and real allocation decisions.

Once the ANN is trained, it can be used to predict the optimal allocation decisions for new input data, which represent the current state of the Fog environment. This enables efficient and effective allocation of resources in a Fog environment, which can improve the performance and reliability of Fog-based applications.

### 4.1. Steps in proposed model

For an optimal utilization of the resources in Fog-Edge computing environment, following key step are followed in ANN inspired Antlion based orchestration in a Fog-Edge computing environment. The high-level block diagram of an ANN inspired Antlion-based Orchestration Algorithm for Fog-Edge Environment includes:

**Input:** Fog-Edge Environment Parameters

1: Initialize the Antlion population with random positions and velocities.

2: For each Antlion, encode its position and velocity using a neural network.

3: Evaluate the fitness of each Antlion based on its position and velocity using the fitness function.

4: Select the Antlion with the highest fitness score as the leader.

5: Update the position and velocity of each Antlion based on the leader’s position and velocity using the Antlion-inspired update rules.

6: For each Antlion, decode its new position and velocity using a neural network.

7: Evaluate the fitness of each Antlion again using the fitness function.

8: Check if the termination condition is met. If so, stop the algorithm and return the solution. If not, go to step 4.

9: Orchestrate the Fog-Edge environment based on the solution obtained from the Antlion-inspired algorithm.

The time complexity of proposed model is divided into two parts training and prediction. The time complexity of prediction is constant “C” as the trained neural network takes constant time to predict the resource (Node where the request will be processed). The proposed model takes most time in dataset preparation and model training which is a one-time process. Dataset preparation using Ant Lion algorithm takes time complexity of O(N) where N is the problem size which is the number of tasks to be scheduled. Where as compared to other existing metaheuristic models inspired from Fuzzy logic O(N^2^), Genetic algorithm O(g(nm + nm + n)) where “g” is the number of generations, “n” is the number of population and “m” ins the population size. and particle swarm optimization is O(nmd) where “n” is the population size, “m” is the number of iteration and “d” is the parameters dimensions. In general existing models are dependent of the number of iteration/evolution taking place or have complexity much higher like N^2^ on the other hand proposed model takes constant time to predict the resource for the schedule.

The over head of the proposed model is the retraining of the trained model in order to overcome the decay of the trained neural network. Decay of the trained model is a phase where the accuracy of the trained model changes with changes in the environment i.e. input. This require retraining training and error back propagation to improve the accuracy of the model. Where the training time complexity is O(Ne * L * E), Ne is the number of neurons, L is number of layers neural architecture and E is the number of samples used for training.

## 5. Experiment and result

**Fig 6:**
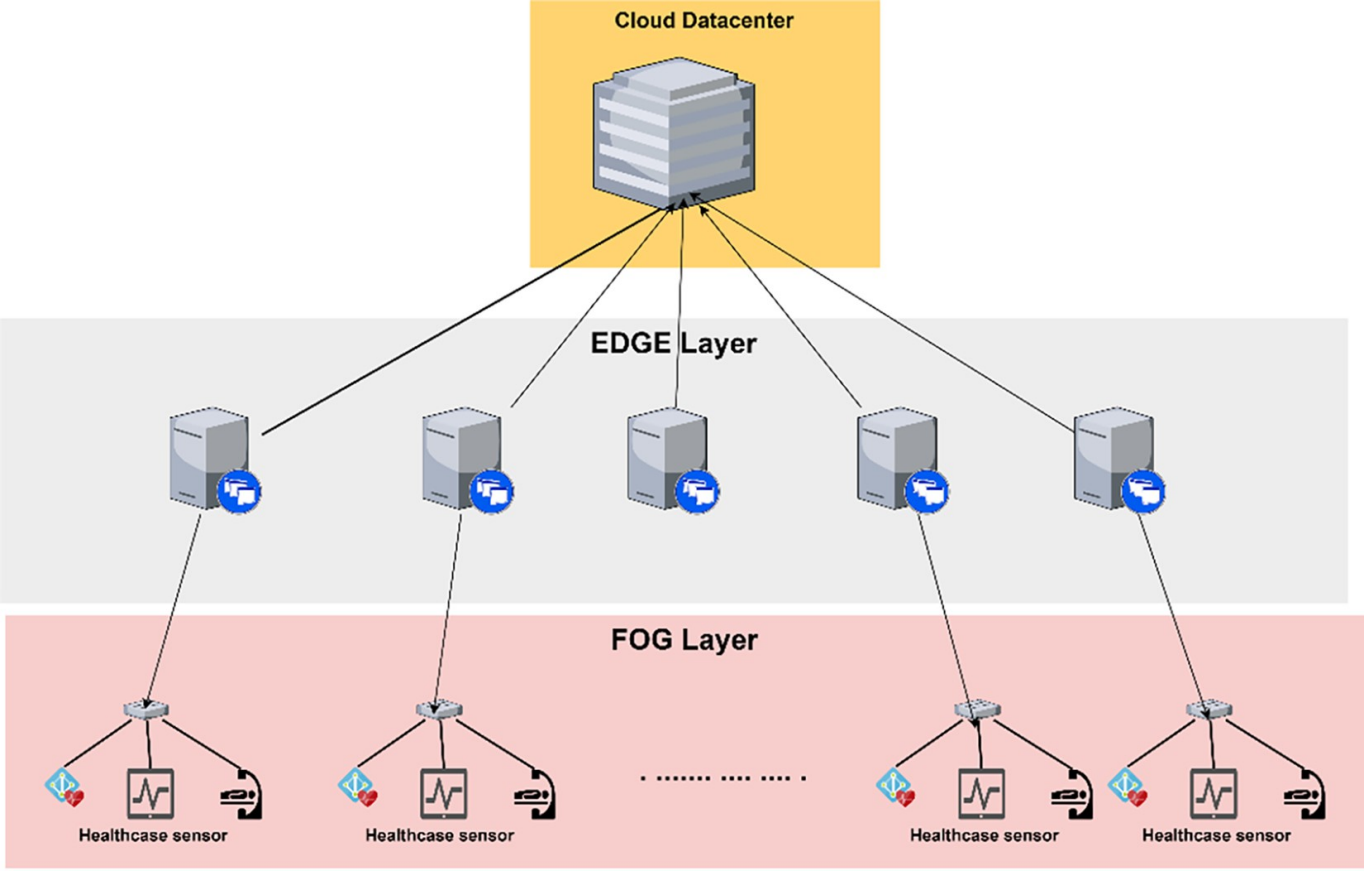
Layer architecture of EDGE computing and healthcare application.

**Fig 7:**
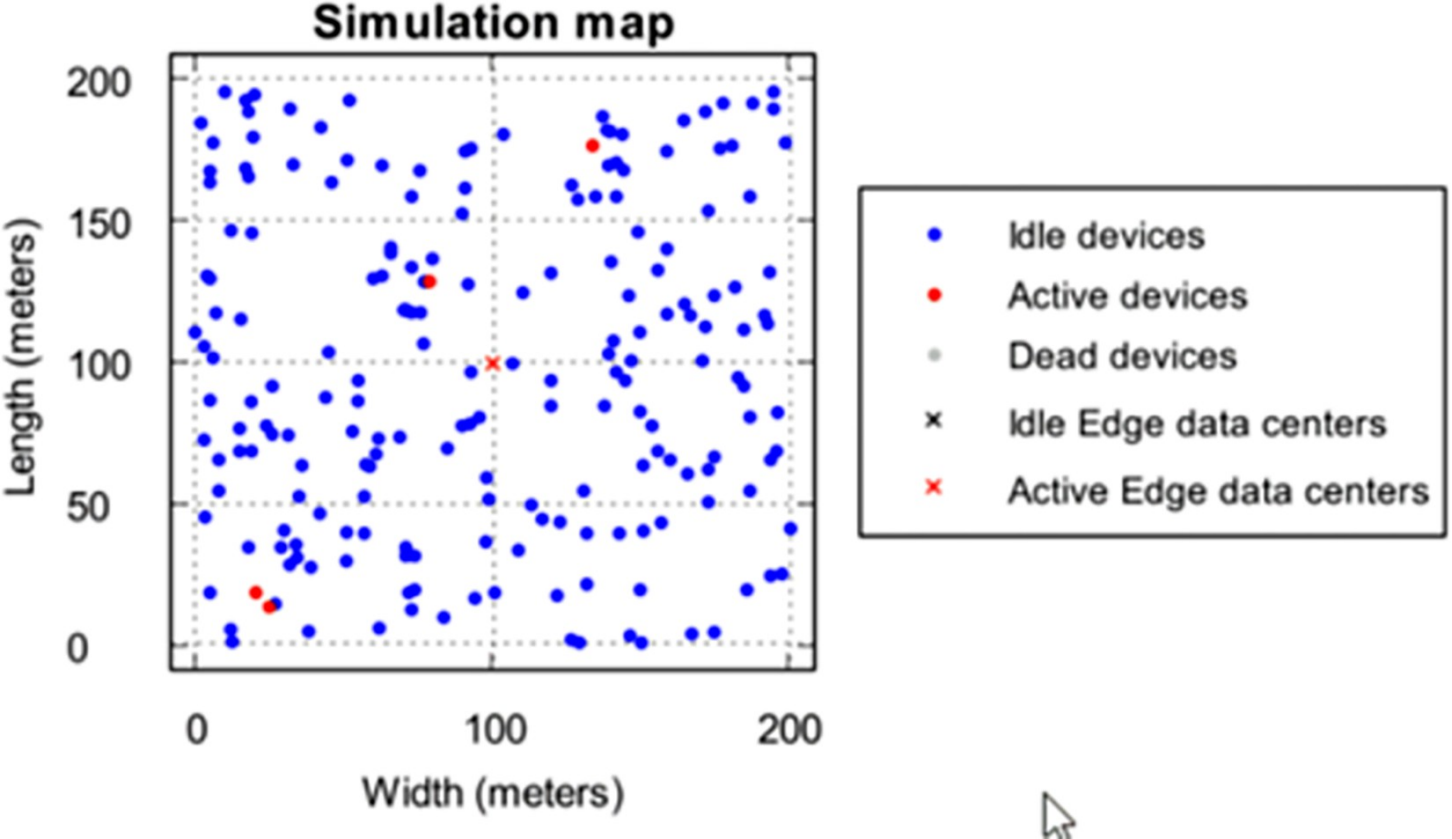
Distribution of Fog and EDGE nodes over an area.

The experiments are performed using PureEdgeSim [24] a well-defined simulation environment for Fog and Edge environment setup. The tool provides configuration to simulate various environments like healthcare, mobile network, vehicular network and many more with clustering and orchestration algorithms. The proposed algorithm is embedded into this tool at orchestrator level for decision making. The experiment is simulated with multiple configurations to study the performance of the proposed and existing model with increasing load and resources. We have chosen several Edge devices ranging from 50 to 300 and 500–3000 to study the performance of the proposed algorithm under overloaded and under loaded conditions. Fig 6 showcase the architecture of the environment for healthcare scenario where Fog nodes are healthcare sensors to generate request, the middle layer is the EDGE layer with EDGE server and cloud datacenter at the top. Table 3 shows the configuration of the cloud and EDGE servers. Table 4 shows the configuration of the Fog node which can generate a computing request for 20% and 50% CPU utilization which defines the Fog node type. The simulation is done for a healthcare application where the number of sensor nodes increases with number of EDGE servers. The simulation consists of Fog nodes spread over an area of 200 * 200 meters where sensor nodes are spread randomly. Fig 7 showcase the spread of nodes over the area. The experiment are performed using variable configuration Edge server and health care application requests. This allows to test the performance of algorithm is more realistic environment with heavy and light computational intensive tasks which are randomly generated.

**Table 3:** Configuration of cloud and EDGE servers.

	Cloud Datacentre	EDGE Server Type 1	EDGE Server Type 2	EDGE Server Type 3
**Core**	200	5	4	8
**MIPS**	40000	20000	4000	10000
**RAM**	16000	8000	4000	4000
**Storage**	1000000	1024000	32000	128000
**Bandwidth (MB)**	10000	5000	4000	5000

**Table 4:** Configuration of Fog node requests.

	Healthcare Sensor node	Healthcare Heavy application
**Task generation rate**	20	3
**CPU utilization %**	20%	50
**Latency (Seconds)**	0.02	300
**Request size (Kilobytes)**	20	2500
**Output size (Kilobytes)**	20	200
**task_length (Million Instruction)**	500	30000
**Bandwidth (MB)**	100	200

The proposed model is tested in two scenarios with load ranging from 50–300 nodes and 500–3000 Edge servers. The number of tasks generated in first scenario id 5000–30500 and for scenario 2 it ranges from 50000–30500 tasks.

This study compares various performance parameters given below for under loaded, average loaded and average load condition. In this section a comprehensive comparative study is showcase where the proposed model is compared with existing models like Fuzzy logic [25, 26], Genetic algorithm [27] and Particle swarm optimization [28, 29].

### 5.1 Energy consumption

We first analyze the energy consumption at various levels. Energy Consumption is the total quantity of energy used at various levels. The energy consumption model of the Edge device is framed as follows: Consider the task *T*_*k*_ placed on the Edge device for execution. The time taken to execute the task is calculated through the load of task *T*_*k*_ and processing power of the Edge device.

$$ET_{A}=\sum_{j=1}^{m}\frac{{LD}_{j}}{P_{E}}$$ (12)

where *LD*_*j*_ is the load of the task *j*, *P*_*E*_ is the processing power of Edge device. The total execution time of all tasks in the Edge device is represented as *ET*_*A*_. Eq (13) represent the energy consumption of an Edge device.

$$E_{edge}={Pow}_{edge}\times ET_{A}$$ (13)

where *Pow*_*edge*_ denotes the power while executing the tasks for Edge device. *E*_*edge*_ represents the energy consumption of the Edge device.

**Fig 8:**
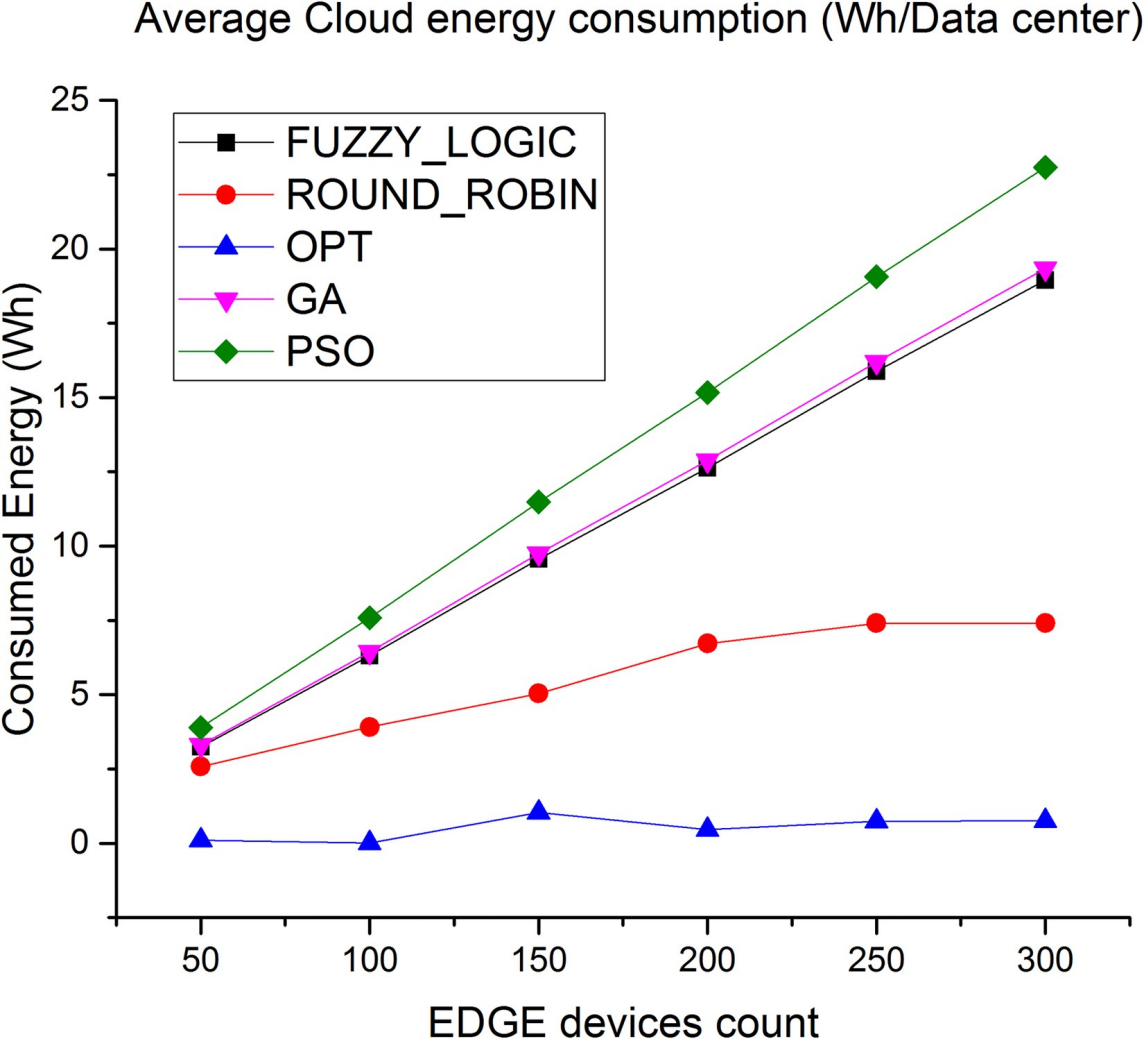
Average energy consumption in cloud.

**Fig 9:**
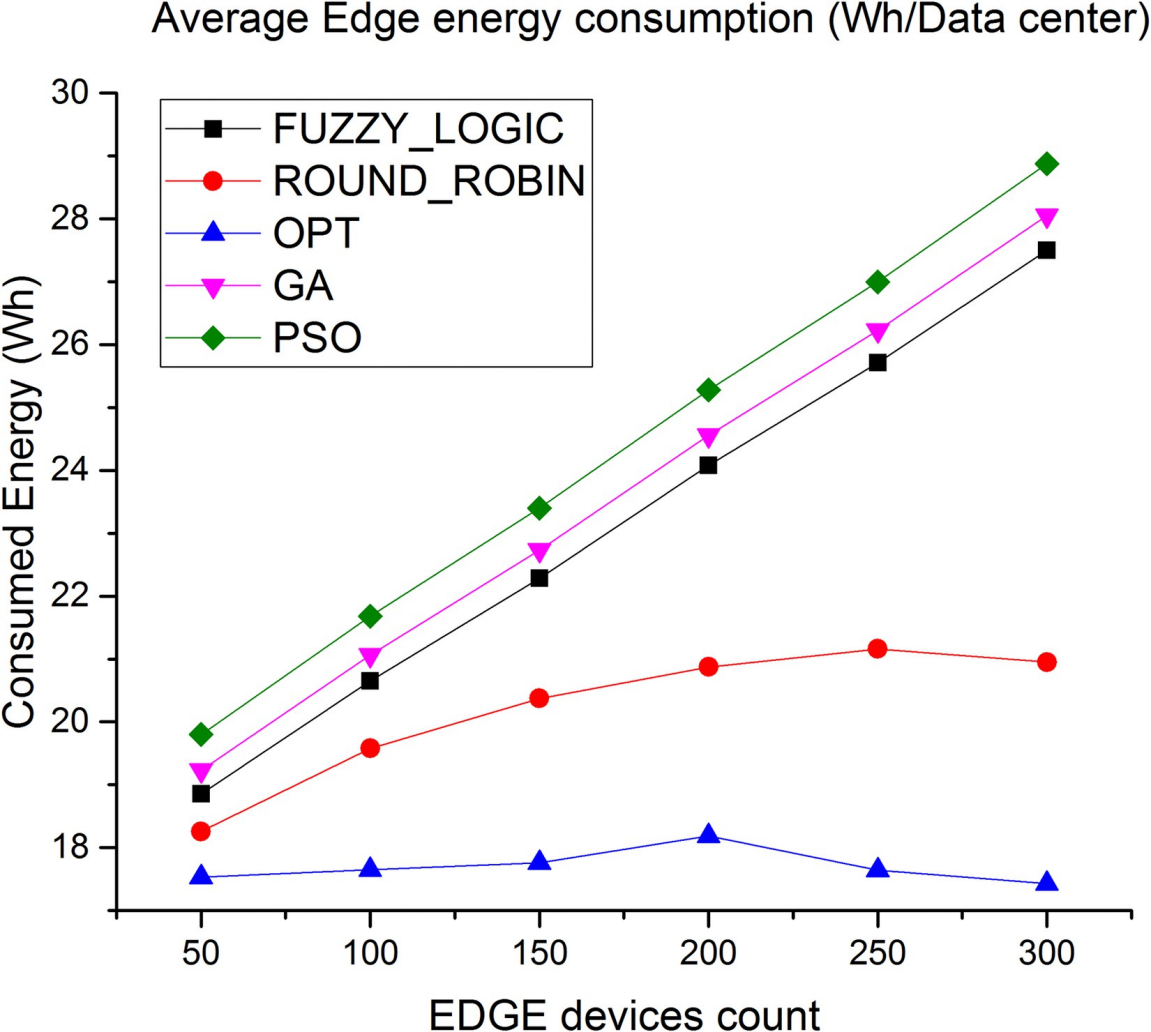
Average energy consumption in Edge.

Fig 8 shows the evaluation result of energy consumption in cloud layer. Average energy consumption via the proposed algorithm is less by 90% compared to fuzzy logic and 60% compared to round robin respectively. Similarly, as shown in Fig 9 average energy consumption in Edge layer less by 30% compared to fuzzy logic and 14% compared to round robin algorithm.

### 5.2 CPU utilization

**Fig 10:**
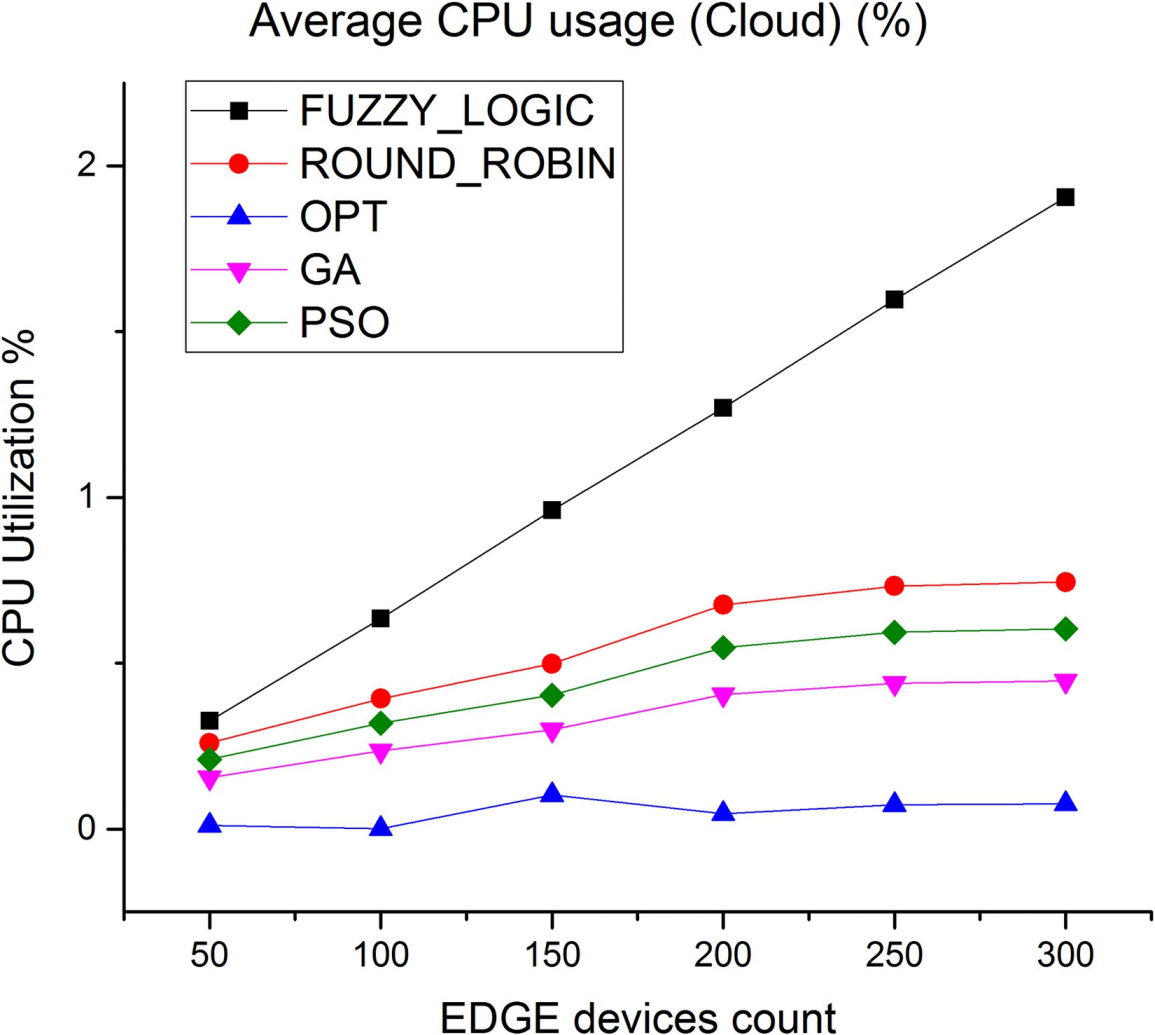
Average CPU utilization in cloud.

**Fig 11:**
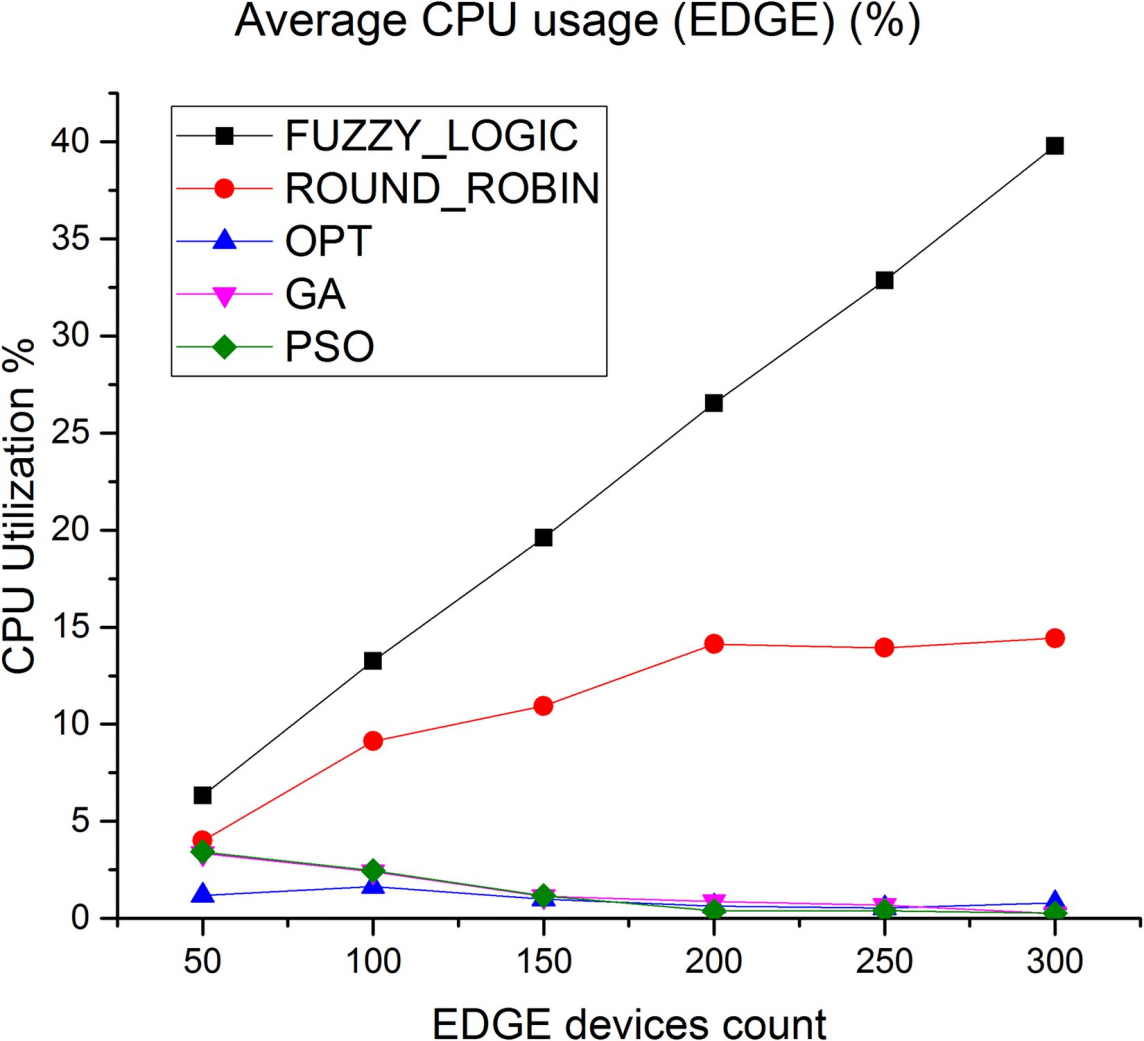
Average CPU utilization in Edge.

CPU utilization is another key parameter we have considered to evaluate the algorithms. Fig 10 shows the average CPU utilization in cloud layer, it is observed that the average CPU utilization rate of the proposed method is higher by 89% compared to fuzzy logic and 50% compared to round robin respectively. Fig 11 depicts the average CPU utilization in Edge layer, here average CPU utilization of the proposed method is higher by 91% compared to fuzzy logic and 85% compared to round robin respectively.

### 5.3 Network utilization

**Fig 12:**
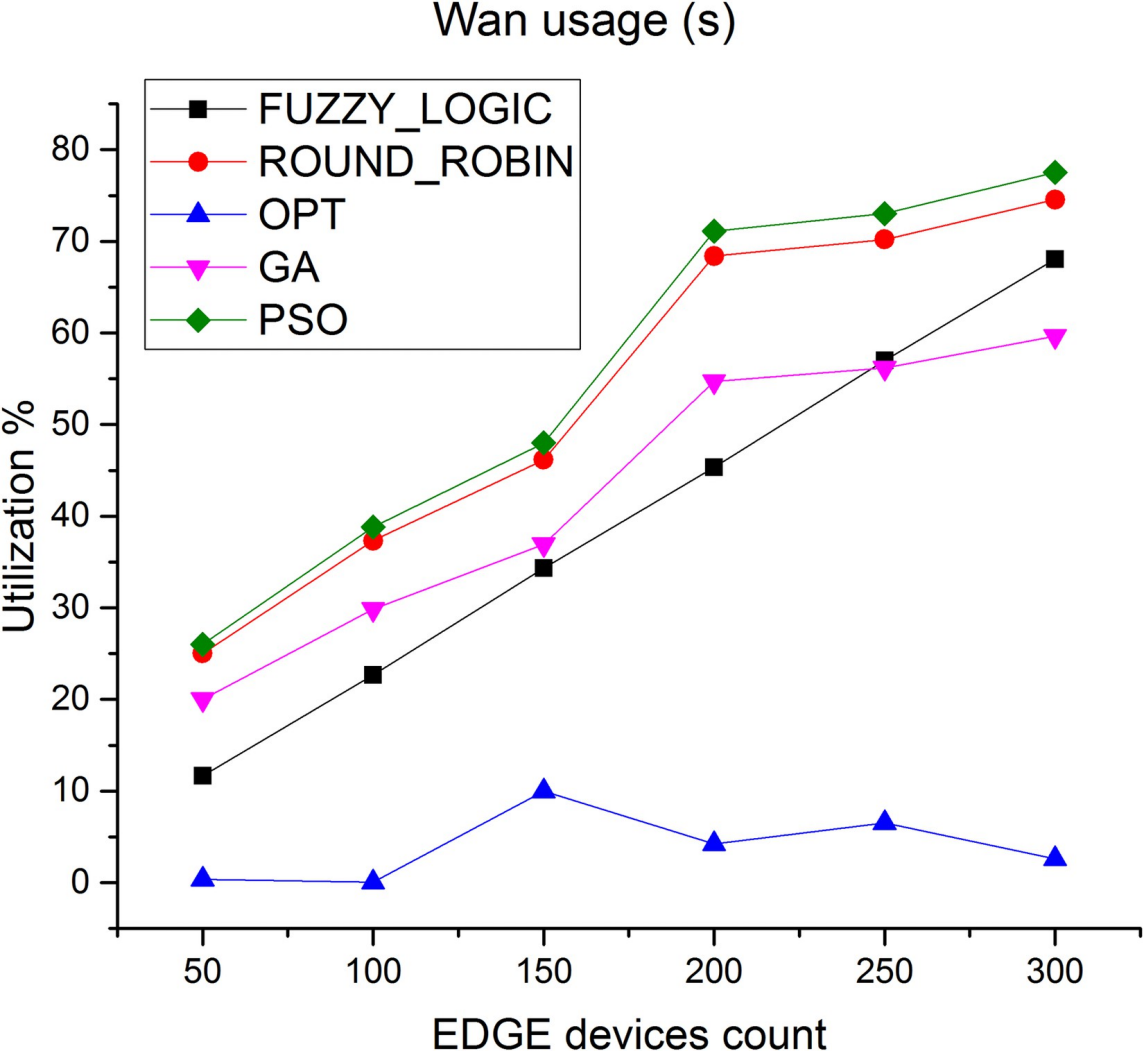
WAN utilization in Edge.

Another parameter incorporated to evaluate the algorithms is network usage. Fig 12 shows the WAN utilization by all the algorithms in the Edge layer. As shown in the figure, average WAN usage of the proposed method is higher by 90% compared to fuzzy logic and 93% compared to round robin respectively.

### 5.4 Waiting time

**Fig 13:**
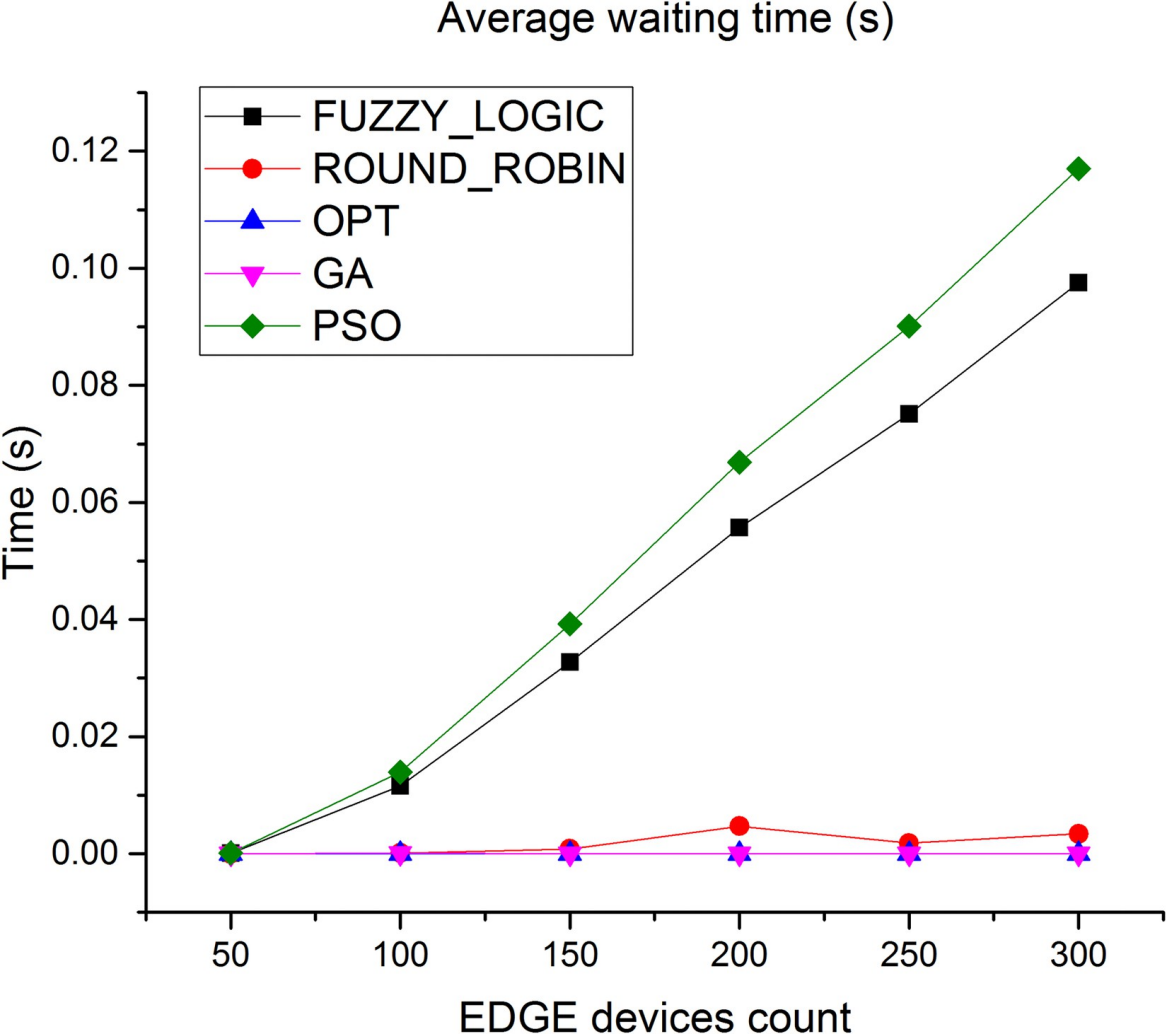
Average waiting time in Edge.

Since the users’ requests are processed at the Edge level, the amount of waiting time required is very much less compared to waiting time at the cloud level. Average waiting time in Edge layer is depicted in Fig 13. As observed, the average waiting time decreases by 96% compared to fuzzy logic and 1.4% compared to round robin respectively.

### 5.5 Tasks completion

**Fig 14:**
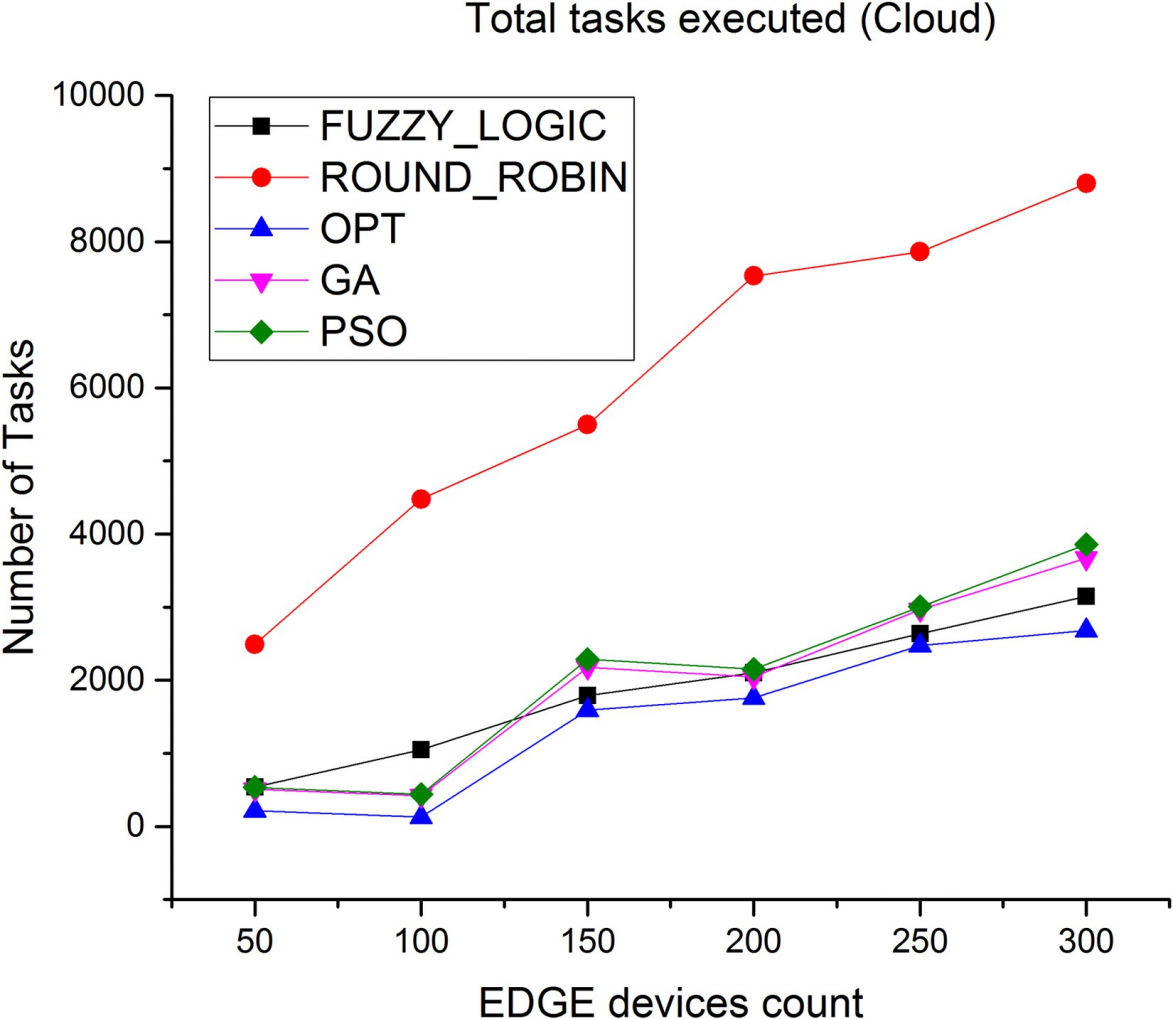
Number of tasks executed in cloud.

**Fig 15:**
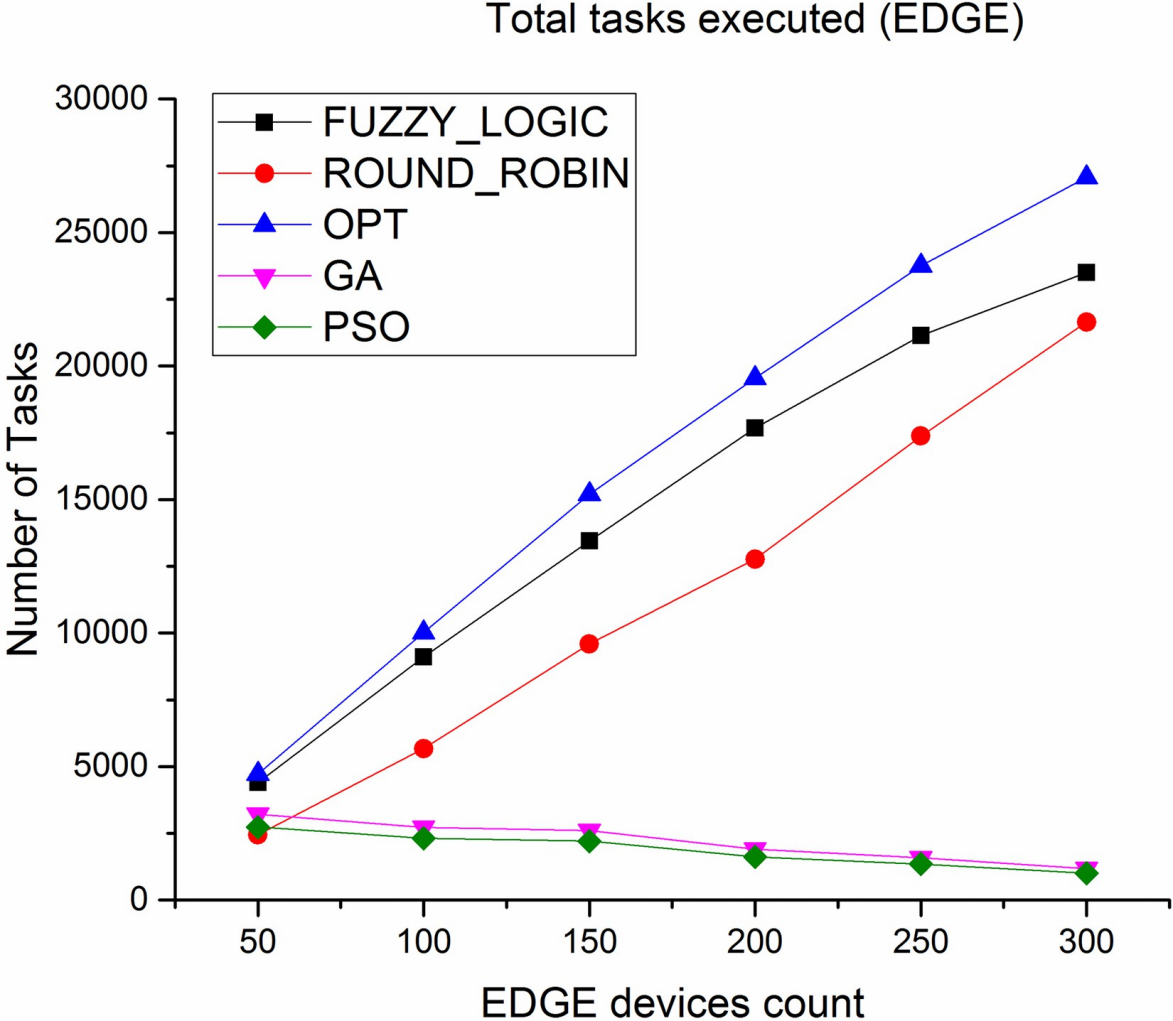
Number of tasks executed in Edge.

Number of tasks successfully executed at cloud and Edge layer is depicted in Figs 14 and 15 Since the Edge is closer to the users, more number of tasks can be completed in a shorter time. As observed, the total number of tasks successfully executed at Edge level is very high compared to number of tasks executed at cloud level, thus the proposed algorithm results in high throughput.

Further the experiment is been conducted over 500–3000 Edge devices to study the performance in real work scenarios of healthcare application where the node definition is showcase in Table 4. The experiments are performed to evaluate energy efficiency, CPU utilization and number of task completed over cloud and edge. The main aim is to study the computational load that is transferred from cloud to Edge servers because the main aim of Edge computing is to compute majority of tasks over the Edge servers with high performance.

**Fig 16:**
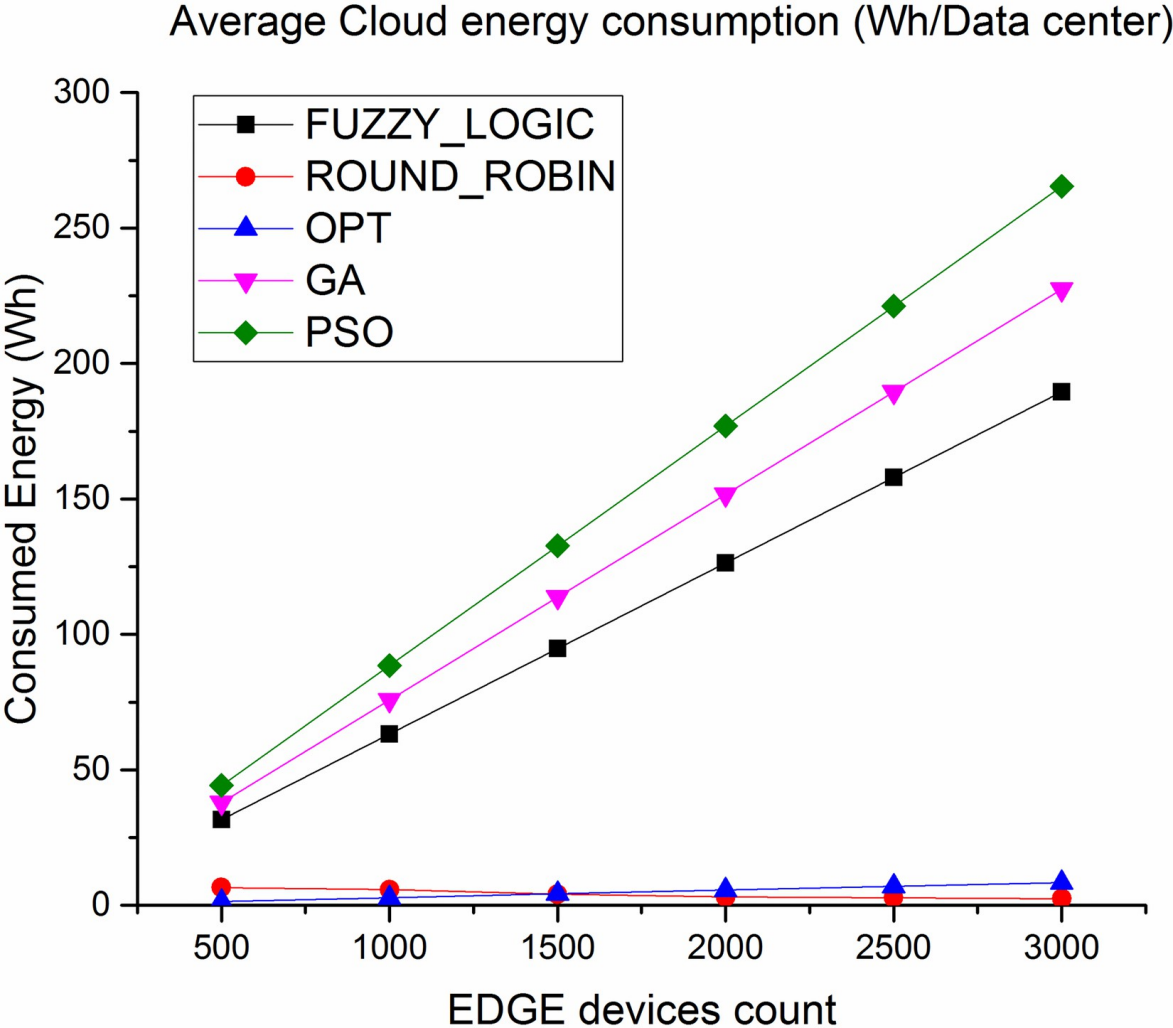
Average energy consumption in cloud.

**Fig 17:**
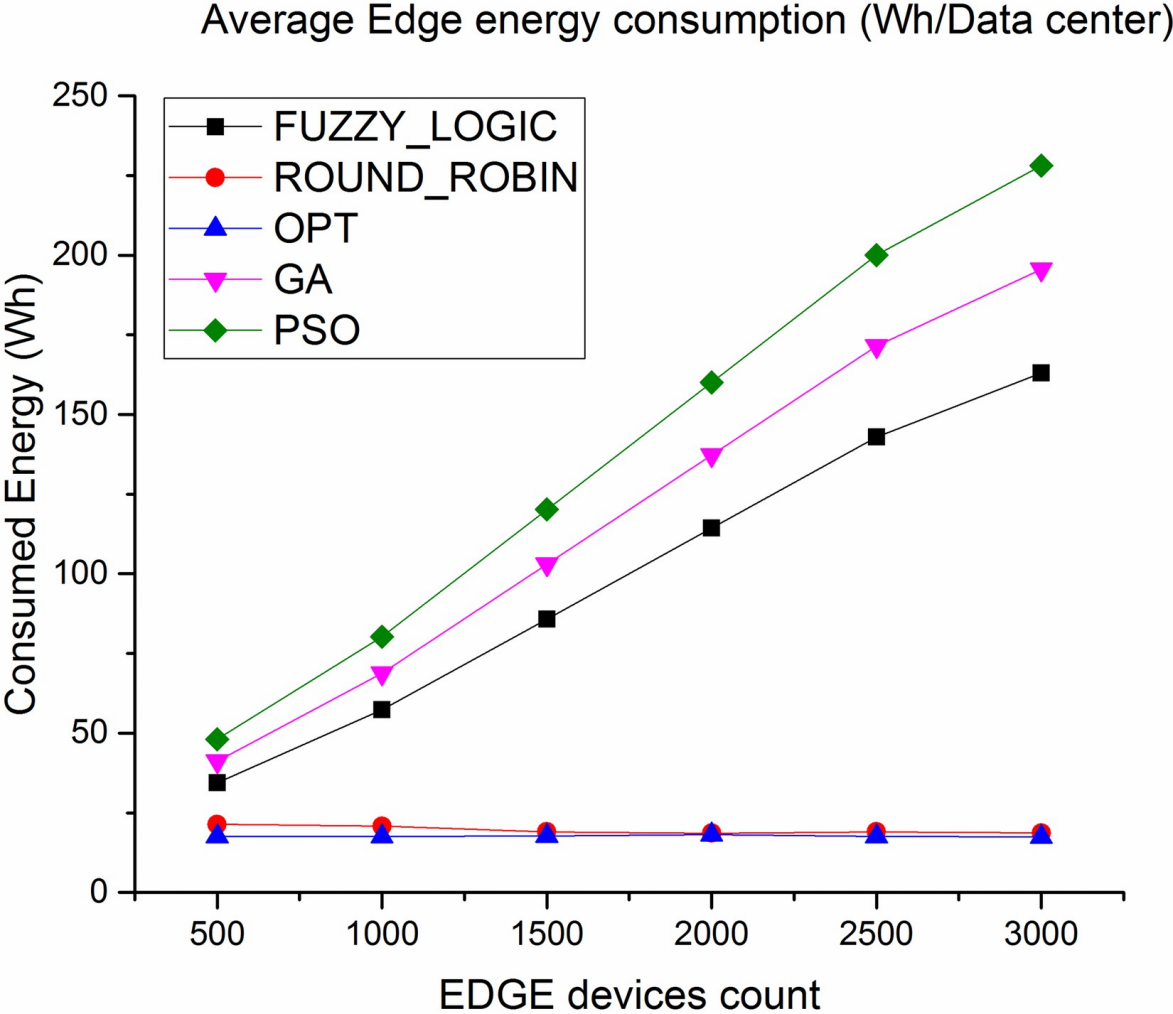
Average energy consumption in Edge.

Figs 16 and 17 showcases the average energy consumption (Wh/Datacenter) where the energy consumed at the cloud datacenter is least because less number of task load is diverted over cloud datacenter. On the other hand the energy consumption of the Edge server is least for proposed algorithm because the average load over the Edge server is high with evenly distributed tasks with no hotspots and under utilized server where as compare to lated Fuzzy logic and genetic approaches are good in balancing the load over the cloud and Edge but fails to evenly distribute the tasks over Edge servers which leads to high energy consumption under over loaded condition. The figure also shows competing performance of Round robin but that is because most of the tasks are not completed due to deadline failure resulting in least energy consumption.

**Fig 18:**
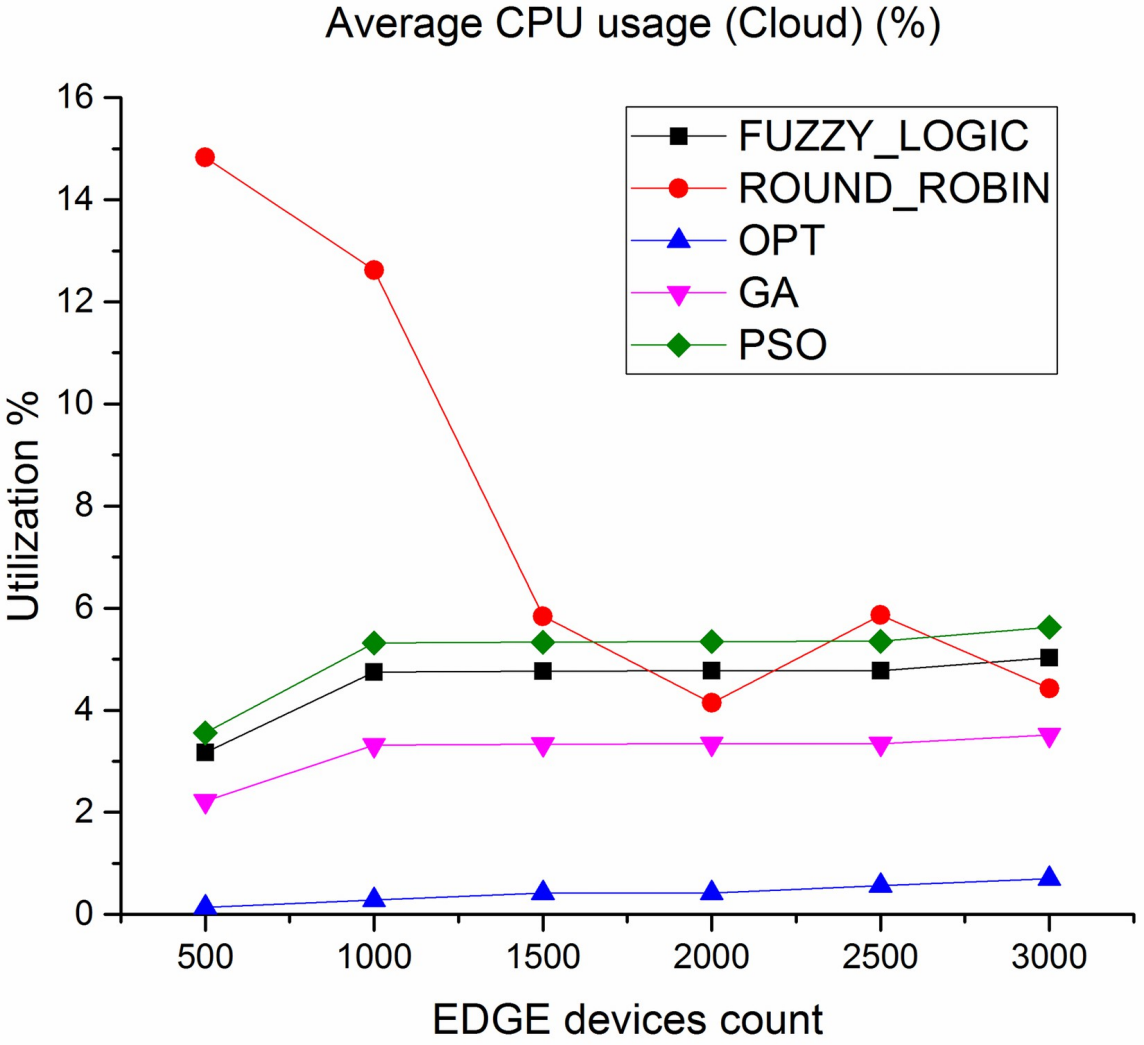
Average CPU utilization in cloud.

**Fig 19:**
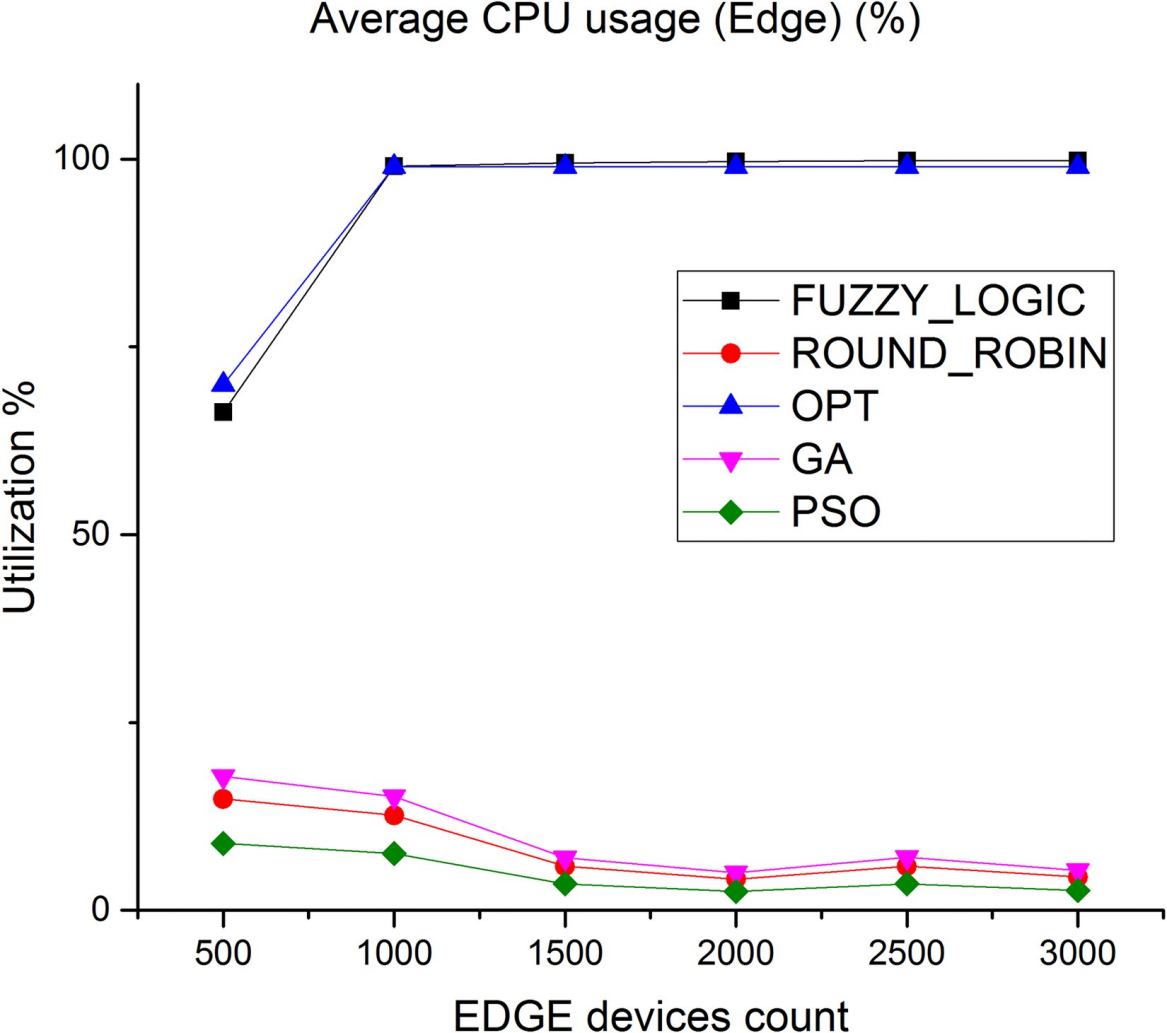
Average CPU utilization in Edge.

**Fig 20:**
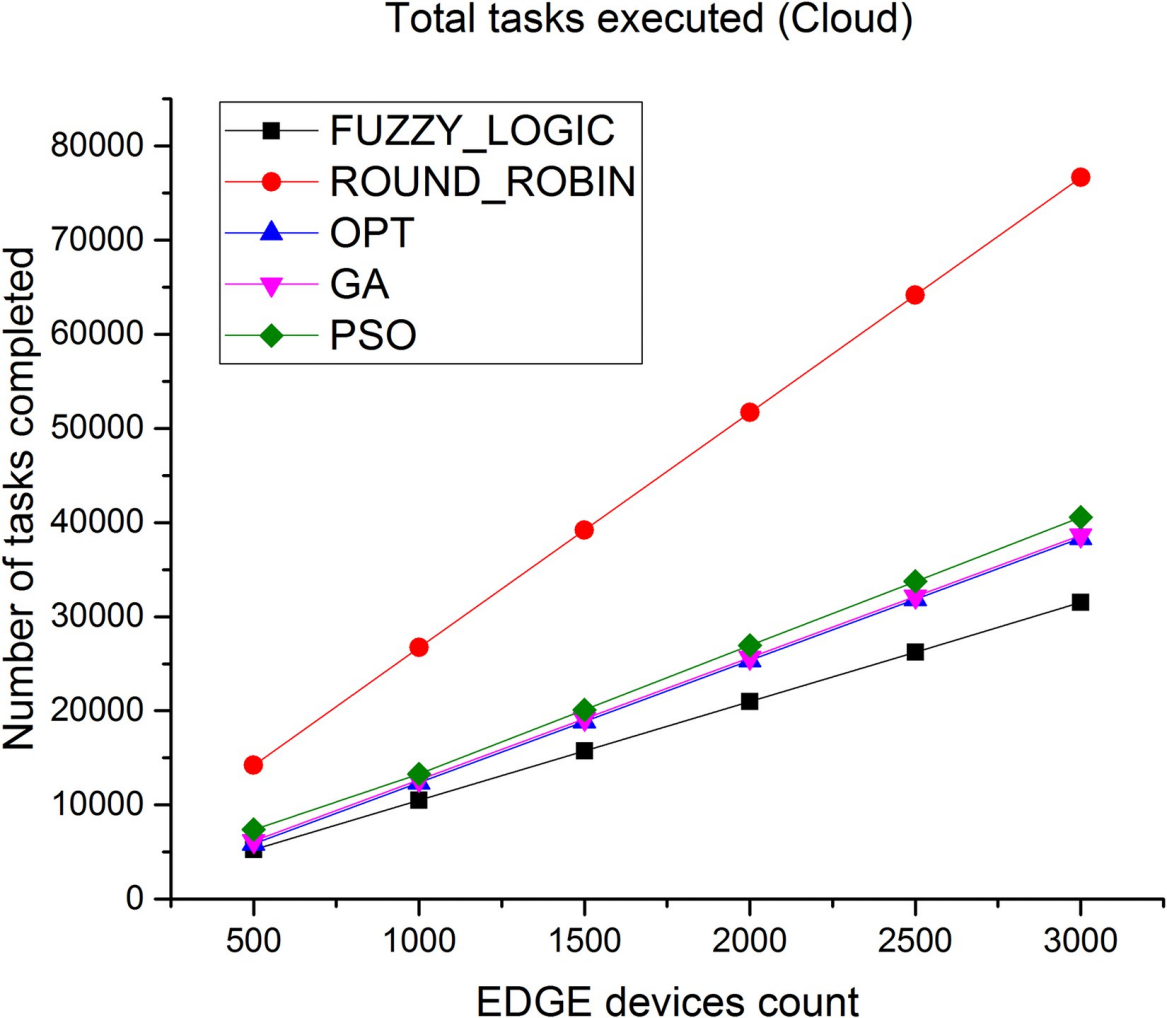
Number of tasks executed in cloud.

**Fig 21:**
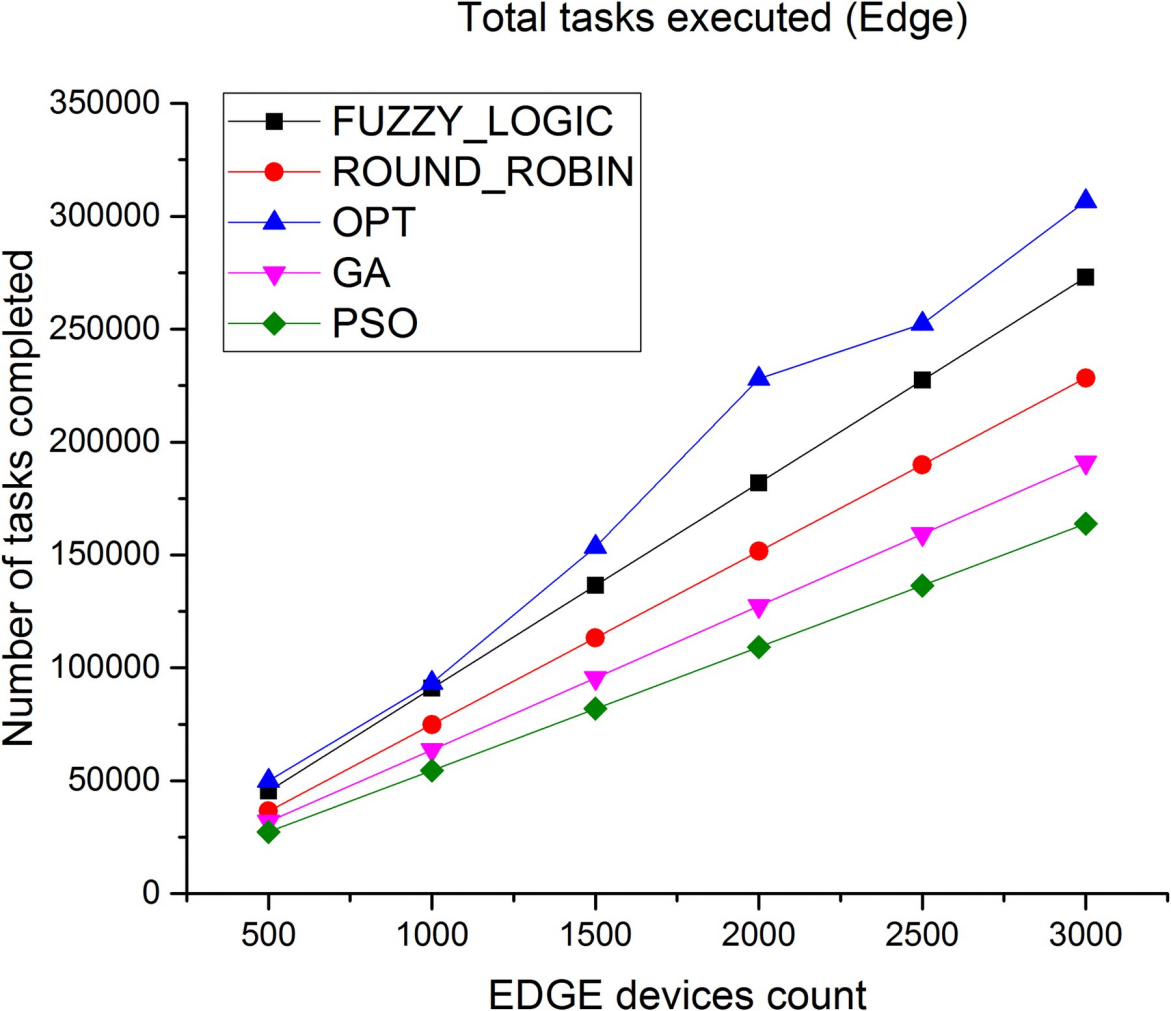
Number of tasks executed in Edge.

Figs 18 and 19 showcases the average CPU utilization in cloud and Edge servers. The results shows the CPU utilization of cloud is least for the proposed algorithm and for Edge servers the average CPU utilization increases to 99% under heavy loaded condition. Similar performance is showcased in Figs 20 and 21 where a study of number of tasks completed over cloud and Edge servers is showcased. Figure shows that majority of tasks are completed over Edge servers as compared to other existing algorithm and to avoid task failure in over loaded condition the tasks are also diverted to cloud servers which increases the number of task completed on cloud when the task load increases.

## 6. Conclusion

The goal of this work is to propose an intelligent orchestration algorithm for the Fog-Edge environment using ANN and Antlion algorithm. The intelligent ANN inspired orchestration approach aims to enhance the performance of the Fog-Edge system while reducing energy consumption and maximizing resource utilization and increase the success rate of the tasks executed tasks with increasing the Edge device count. To evaluate the algorithm, various scenarios considering 50 to 300 Edge devices are tested. The experimental results show that the average cloud energy consumption improvement of 95.94%, and average Edge energy consumption improvement of 16.79%, 19.85% in average CPU utilization in Edge computing environment, 10.64% in average CPU utilization in cloud environment, and 23.33% in average network utilization, and the average waiting time decreases by 96% compared against the fuzzy logic approach, and 1.4% compared to round robin approach respectively. In addition, the ANN inspired Antlion based orchestration technique increases the average WAN utilization by 91.5% in the Edge level compared to other algorithms. The proposed algorithm significantly improves performance metrics including throughput of the Fog-Edge system and reduces the waiting time to process a given task. In future, the experiment will be carried out using other hybrid approaches considering real cloud scenario with cost and service level agreement parameters for evaluation.
